# Loss-of-function mutations in the histone methyltransferase EZH2 promote chemotherapy resistance in AML

**DOI:** 10.1038/s41598-021-84708-6

**Published:** 2021-03-12

**Authors:** Julia M. Kempf, Sabrina Weser, Michael D. Bartoschek, Klaus H. Metzeler, Binje Vick, Tobias Herold, Kerstin Völse, Raphael Mattes, Manuela Scholz, Lucas E. Wange, Moreno Festini, Enes Ugur, Maike Roas, Oliver Weigert, Sebastian Bultmann, Heinrich Leonhardt, Gunnar Schotta, Wolfgang Hiddemann, Irmela Jeremias, Karsten Spiekermann

**Affiliations:** 1 Department of Medicine III, University Hospital, LMU Munich, Munich, Germany; 2 Department of Biology II and Center for Integrated Protein Science Munich (CIPSM), Human Biology and BioImaging, LMU Munich, Planegg Martinsried, Germany; 3 Research unit Apoptosis in Haematopoietic Stem Cells (AHS), Helmholtz Zentrum München, Munich, Germany; 4 Center for Human Genetics and Laboratory Diagnostic (AHC), Martinsried, Germany; 5 Biomedical Center and Center for Integrated Protein Science Munich, LMU Munich, Martinsried, Germany; 6 German Cancer Consortium (DKTK), Heidelberg, Germany; 7 Department of Pediatrics, Dr. von Hauner Children’s Hospital, LMU Munich, Munich, Germany; 8 Anthropology and Human Genomics, Department of Biology II, Ludwig-Maximilians-University, Martinsried, Germany; 9 German Cancer Research Center (DKFZ), Heidelberg, Germany

**Keywords:** Experimental models of disease, Acute myeloid leukaemia

## Abstract

Chemotherapy resistance is the main impediment in the treatment of acute myeloid leukaemia (AML). Despite rapid advances, the various mechanisms inducing resistance development remain to be defined in detail. Here we report that loss-of-function mutations (LOF) in the histone methyltransferase EZH2 have the potential to confer resistance against the chemotherapeutic agent cytarabine. We identify seven distinct EZH2 mutations leading to loss of H3K27 trimethylation via multiple mechanisms. Analysis of matched diagnosis and relapse samples reveal a heterogenous regulation of EZH2 and a loss of EZH2 in 50% of patients. We confirm that loss of EZH2 induces resistance against cytarabine in the cell lines HEK293T and K562 as well as in a patient-derived xenograft model. Proteomics and transcriptomics analysis reveal that resistance is conferred by upregulation of multiple direct and indirect EZH2 target genes that are involved in apoptosis evasion, augmentation of proliferation and alteration of transmembrane transporter function. Our data indicate that loss of EZH2 results in upregulation of its target genes, providing the cell with a selective growth advantage, which mediates chemotherapy resistance.

## Introduction

Acute myeloid leukaemia (AML) is a heterogeneous haematological malignancy, characterised by clonal expansion of abnormal, undifferentiated myeloid precursor cells. Even though many patients with AML respond well to induction chemotherapy, relapse and refractory disease are common, representing the major cause of treatment failure. Treatment with cytarabine (AraC) and daunorubicin (DNR) remains the standard care for AML patients, although several new therapeutic strategies have been implemented within the last years^1–3^. Epigenetic dysregulation of DNA methylation or histone modifications has been identified in many malignant tumors^4,5^ and can be considered as a cause of cancer development and progression^6,7^. Since considerable insight concerning those epigenetic changes has been gained in recent years, many therapy concepts targeting the involved regulatory factors have been proposed and hold promise for novel treatment approaches^8,9^.

Enhancer of zeste homolog 2 (EZH2) is a lysine methyltransferase found as the central core protein of the polycomb repressive complex 2 (PRC2)^10^. Comprising four subunits (SUZ12, EED, EZH2/EZH1 and RbAp46), this complex mediates transcriptional repression by catalysing the trimethylation of histone H3 at lysine 27 (H3K27me3)^11^. EZH2 has been found to serve a dual purpose, as either tumour suppressor or oncogene, depending on the type of cancer^12–17^. In leukaemia, overexpression of EZH2 has been observed in CLL^18^, paediatric T-ALL^19^ and CML^20^, while other studies reported EZH2 levels to be decreased in CMML^21^ as well as ALL^18,19,22^. A recent study of Basheer et al. suggests opposing roles of EZH2 in initiation and maintenance of AML^23^.

EZH1, an EZH2 homolog capable of partially compensating EZH2 function, holds an essential role in preserving pathological stem cells^24^. Therefore, it might contribute to the already complex role of EZH2 in hematopoietic malignancies^25–27^. Although EZH2 loss-of-function mutations seem to be rare in AML^28^, loss of EZH2 by other mechanisms have been frequently reported and appear to play a major role in disease progression^29,30^. Absence of EZH2 in leukaemia cells was recently found to aberrantly activate BCAT1, resulting in enhanced mTOR signaling^27^ and activation of the oncogene Hmga2 by causing an epigenetic switch from H3K27 trimethylation to H3K27 acetylation^31^. Furthermore, reduced disease-free survival was found to be associated with EZH2 mutations in myeloid malignancies^28,32,33^ including AML^23^. In addition, chemoresistance was found in a recent study on AML patients with poor prognosis and downregulated EZH2^34^.

In our previous study^35^, examining diagnosis/relapse pairs of 50 cytogenetically normal (CN) AML patients, we found mutations in epigenetic modifiers, including EZH2, frequently gained at relapse, suggesting epigenetic mechanisms to be involved in disease progression in a subset of patients. The current study aims to evaluate the importance of chemotherapy resistance in AML. We investigated *EZH2* mutations and their functional loss of methyltransferase activity using patient samples, *in vivo* and *in vitro* patient-derived xenografts (PDX), and haematopoietic cell lines. We found *EZH2* loss-of-function mutations to be involved in the development of resistance against cytarabine and observed upregulation of EZH2 target genes due to loss of H3K27 trimethylation.

## Results

### Recurrent *EZH2* mutations at diagnosis

**Figure 1:**
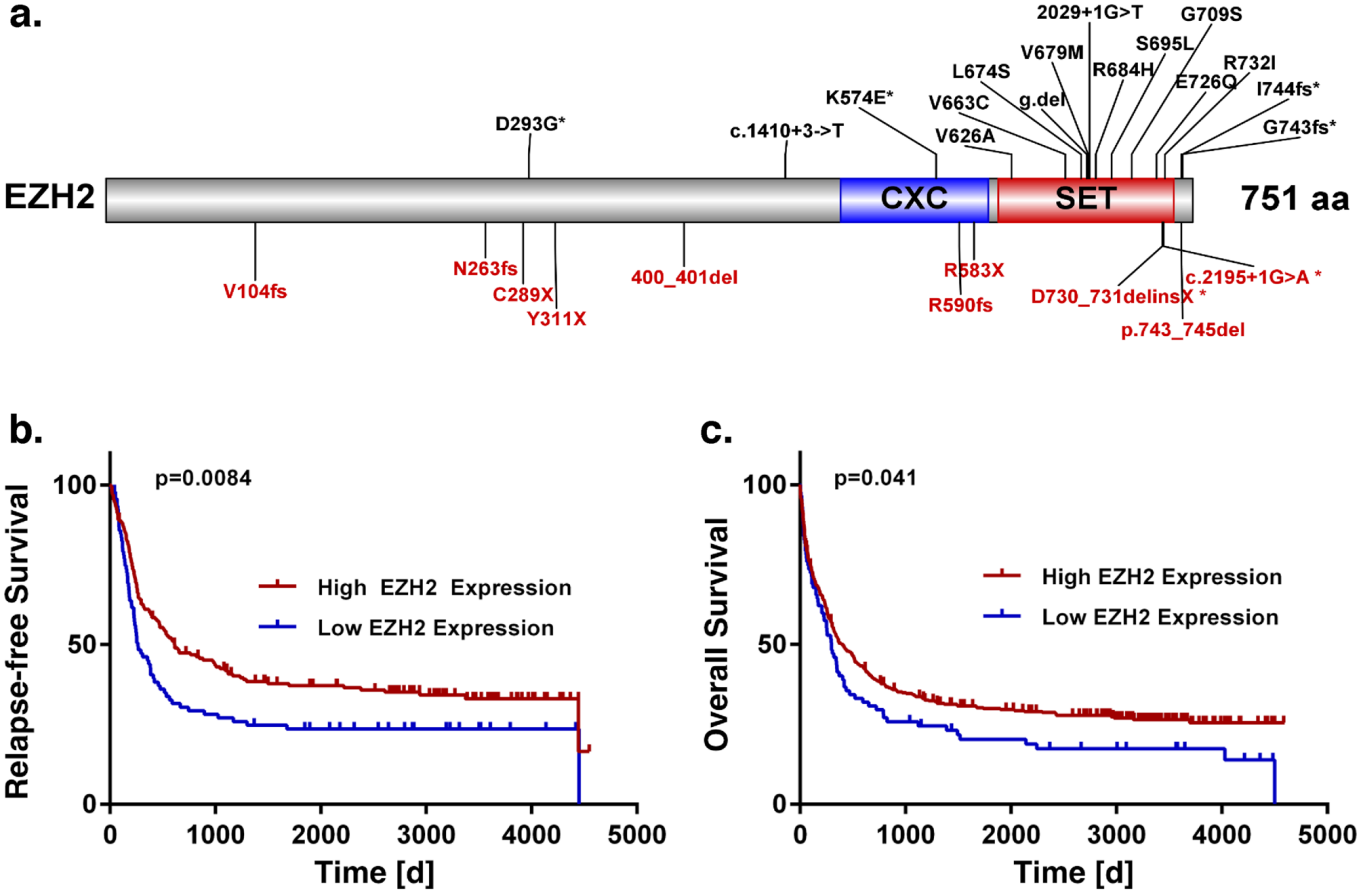
Recurrent *EZH2* mutations. **(a)** Schematic overview of EZH2 protein structure (NM_004456.4) and identified mutations (27 in total, c.2195+1G>A appeared twice) in a cohort of 664 AML patients at diagnosis. Functional domains are indicated at distinct locations and truncating mutations are displayed in red. Patients from Metzeler et al. 2016 (AMLCG-1999, AMLCG-2008). **(b–c)** Survival analysis of patients with low or high *EZH2* mRNA expression at the time point of diagnosis. *EZH2* high and low groups defined by the upper and lower quartile of *EZH2* mRNA expression, independent of mutation status. **(b)** Relapse-free survival (RFS). **(c)** Overall survival (OS). Patients from AMLCG 1999 (GSE37642), n = 517. 21 patients harboured an *EZH2* mutation. P-value calculated by log-rank test.

In our previous work, we analysed 664 AML patients to study recurrently mutated genes, including *EZH2*^36^. In this cohort, 25 patients (4 %) carried an *EZH2* mutation at the time of diagnosis (27 mutations in total, Fig. 1a). Most of these mutations (n = 20, 74%) were located in the SET ([Su(var)3-9, Enhancer-of-zeste and Trithorax]) or CXC (cysteine-rich region, sometimes referred to as pre-SET) domain at the C-terminus of the protein and are responsible for the catalytic activity of the methyltransferase. Furthermore, 41% (11) of mutations cause a stop-gain or frameshift, resulting in a truncated protein. An additional two frameshift mutations result in an elongated protein variant. Mutations most frequently co-occurring with mutated *EZH2* were found in *RUNX1*, *ASXL1*, *DNMT3A* and *TET2* (44%, 40%, 20% and 20%, Supplementary Fig. 1a). Additionally, *RUNX1* and *ASXL1* mutations were found to occur more often in *EZH2* mutated patients (44% and 40%) than in *EZH2* wild type patients (14% and 10%, *p* = 4.6e−04, and *p* = 9.3 e−05, Fisher's Exact Test). In contrast, NPM1, the most frequently mutated gene in our cohort, was found to be mutated less often in *EZH2* mutated (12%) than in *EZH2* wild type patients (34%) (*p* = 2.8 e−02, Fisher's Exact Test). Interestingly, *KDM6A* and *EZH2* mutations were found to be mutually exclusive. Most patients with *EZH2* mutations (76%, n = 19) can be assigned to the adverse risk group (Supplementary Fig. 1a), according to the recent ELN classification^37^.

In order to evaluate the prognostic importance of *EZH2,* we examined the survival of patients dependent on their *EZH2* mutation and expression status. The overall survival (OS) of patients harbouring *EZH2* mutations did not differ significantly from patients without mutation (Supplementary Fig. 1b). However, low *EZH2* mRNA expression was significantly associated with poor relapse-free survival (RFS) and OS in publicly available independent data sets of the AMLCG 1999 trial (GSE37642, Fig. 1b-c) and HOVON (GSE14468, Supplementary Fig. 1) study groups^38–40^. Additionally, monosomy 7, resulting in reduced *EZH2* expression, was associated with poor overall survival (Supplementary Fig. 1c).

### Relevance of EZH2 status in AML relapse

**Figure 2:**
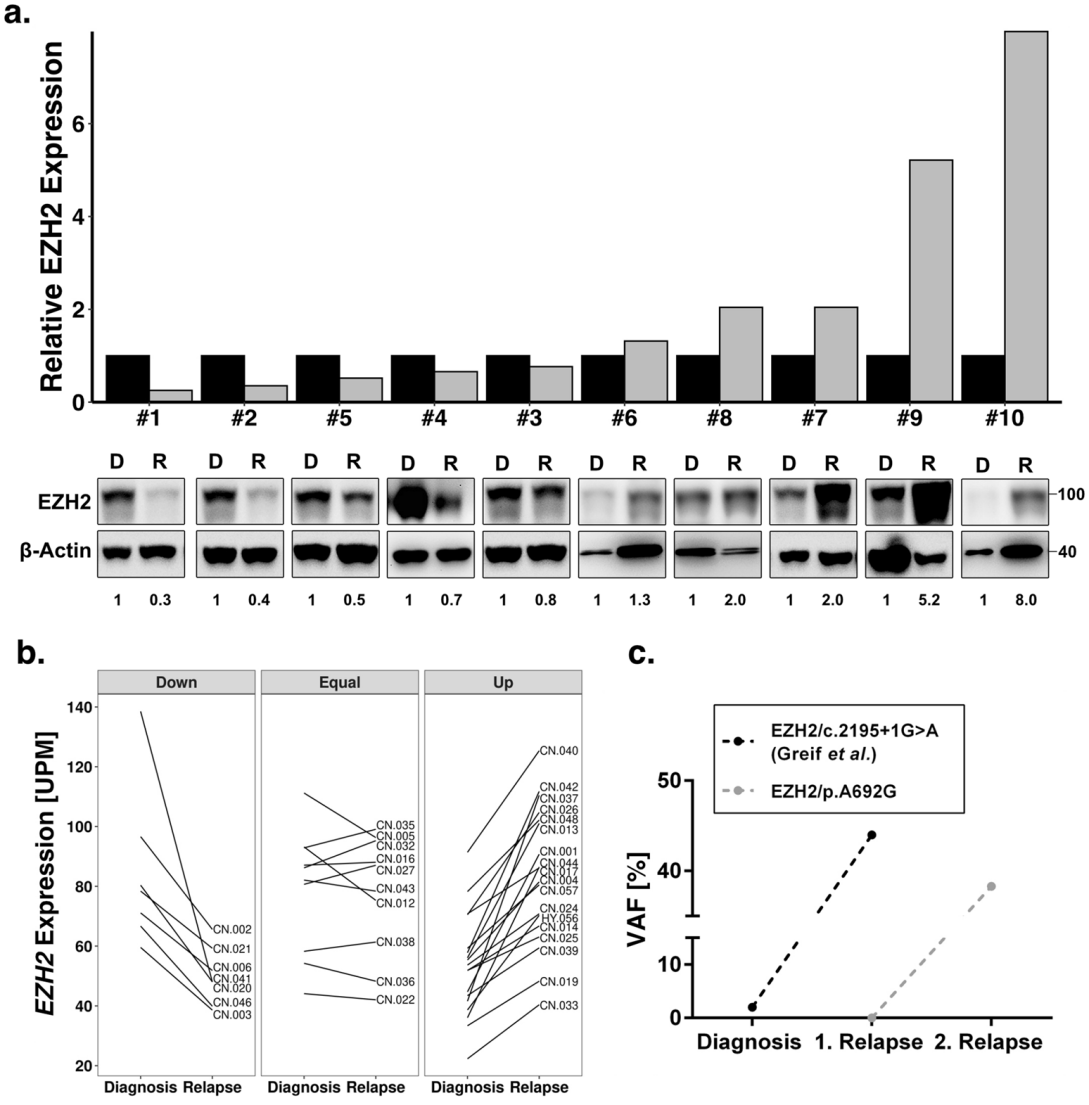
Relevance of EZH2 status in AML relapse. **(a)** Immunoblot for EZH2 protein expression in 10 AML patients at diagnosis and relapse. MW, molecular weight; β-actin, loading control. The ratio of EZH2 to β-actin expression is indicated below and presented in the histogram above. Each relapse value was normalized to the corresponding diagnosis sample. None of the patients carried an *EZH2* mutation. **(b)** *EZH2* mRNA expression between diagnosis and relapse of 32 CN-AML patients from Greif et al. cohort^35^. Up and down are defined as a change in mRNA expression of at least 20%. Three patients carried an *EZH2* mutation. **(c)** Variant allele frequency of the two relapse-associated *EZH2* mutations with outgrowth in first and second relapse.

To further investigate the poor survival in patients with low *EZH2* mRNA expression, we compared protein expression in a set of matched diagnosis and relapse pairs of ten AML patients without *EZH2* mutations (Supplementary Table 2). In 50% of patients, we observed decreased levels of EZH2 protein expression, whereas the other half revealed increased protein expression levels in relapse (Fig 2a). An increase of at least 2-fold in protein expression was found in four patients, whereas a strong decrease (2-fold or more) in protein expression was observed in three patients. An additional analysis of *EZH2* mRNA expression in 32 CN-AML patients revealed a similar heterogenous picture. Downregulation of *EZH2* was found in 22% of patients, while upregulation was found in 53% (Fig. 2b). Additionally, we identified two relapse-associated *EZH2* mutations. EZH2/p.A692G found in the second relapse of patient CN-021 from the Greif et al. cohort^35^ and EZH2/Y733LfsX6 found in the first relapse of a patient from the AML-CG cohort. Both mutations revealed subclonal outgrowth during the course of treatment and increasing variant allele frequencies (VAFs) in relapsed patients (Fig. 2c). Additionally, we found an increase of VAFs in the relapse of three other EZH2 mutations found in the Greif et al. cohort^35^ (Supplementary Fig. 2).

### Functional characterisation of *EZH2* mutations

**Figure 3:**
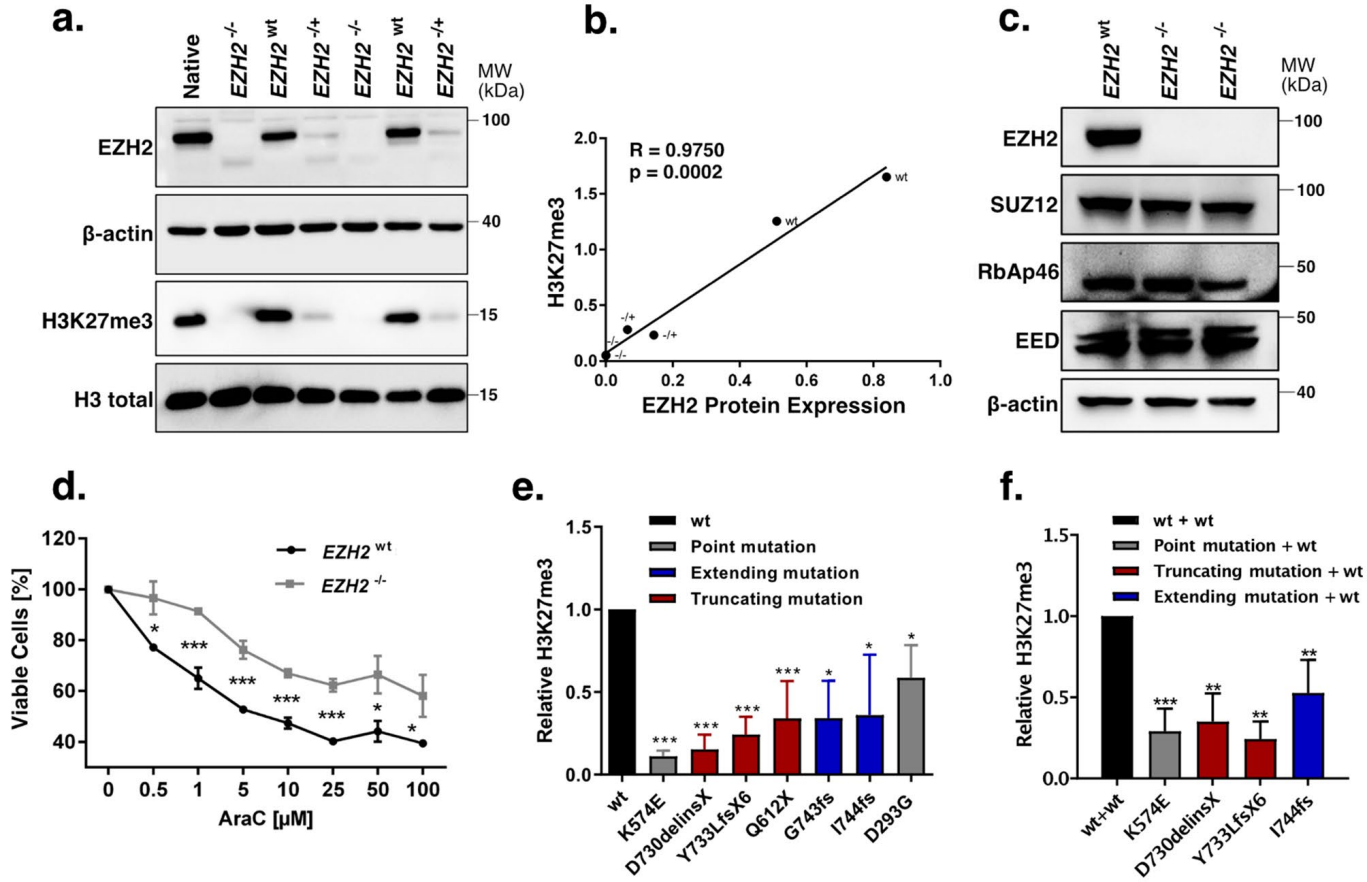
Evaluation of *EZH2* mutations found in AML patients at diagnosis. **(a)** Comparison of EZH2 expression and global H3K27me3 between *EZH2*^*−/−*^, *EZH2*^*+/−*^ and *EZH2*^*wt*^ sc clones in 293T cells. MW, molecular weight. β-actin and H3 total, loading controls. **(b)** Correlation between EZH2 protein expression and global H3K27me3 in 293T sc clones. Pearson's correlation. **(c)** Immunoblot for EZH2, SUZ12, RbAP46 and EED expression in 293T/*EZH2*^*−/−*^ sc clones. MW, molecular weight; β-actin, loading control. **(d)** AraC resistance in one 293T/*EZH2*^*−/−*^ and one 293T/*EZH2*^*wt*^ sc clone. Cells were treated for 72 h with different concentrations of AraC. Viable cells relative to untreated control. **(e)** H3K27me3 levels after re-expression of seven *EZH2* mutations, detected in patient diagnosis samples. Colours referring to protein structural changes caused by the mutation. 293T/*EZH2*^*−/−*^ cells were transfected transiently with *EZH2* constructs 72 h before protein isolation, and global H3K27me3 was evaluated by immunoblot. Values relative to the wild type. **(f)** H3K27me3 levels after re-expression of four *EZH2* mutants in combination with wild type *EZH2*. 293T/*EZH2*^*−/−*^ cells were transfected transiently with *EZH2* wildtype and *EZH2* mutant constructs 72 h before protein isolation. Values relative to the wild type. Unpaired, two-tailed Student’s t-test; *p < 0.05; **p < 0.01; ***p < 0.001. Error bars indicate mean ± s.d of at least three independent experiments.

To evaluate the biochemical activity of the EZH2 variants, we measured global H3K27 trimethylation levels in a 293T/*EZH2*^*−/−*^ model (Fig. 3a), which was established through CRISPR/Cas9 mediated genome editing, targeting exon 3 of *EZH2.* We found EZH2 protein expression levels to be strongly correlated with global H3K27me3 levels (Fig. 3b). In fact, global H3K27me3 was not detectable in any of the tested *EZH2*^*−/−*^ clones, whereas *EZH2*^+/−^ clones showed decreased EZH2 expression as well as reduced global H3K27me3 levels (Fig. 3a). Since EZH2 is only one part of the PRC2 complex, we additionally analysed protein expression of the remaining components SUZ12, RBAP46 and EED. We could not detect aberrant expression of these subunits in both 293T/*EZH2*^*−/−*^ clones (Fig. 3c). Interestingly, both clones showed an increased resistance against AraC compared to the wild type clones (Fig. 3d, Supplementary Fig. 3a) and a slightly reduced colony count was observed in a colony formation assay (Supplementary Fig. 3c). Re-expression of seven different EZH2 variants, (Supplementary Fig. 3b) found in the AML-CG-1999 and AML-CG-2008 studies, could only partially rescue global H3K27me3 levels, indicating a LOF phenotype, while the re-expressed wildtype protein was able to restore complete activity (Fig. 3e).

In addition, co-expression of these variants with wild type *EZH2* led to a reduction of H3K27me3 levels in four mutations, suggesting a dominant-negative effect (Fig. 3f).

To validate the robustness of our 293T/*EZH2*^*−/−*^ model, we performed the rescue experiment with two previously described EZH2 variants. EZH2/p.Y646N, a gain-of-function mutation found in lymphomas^16,17^ and EZH2/Y731, a LOF mutation^41^. We were able to verify the functions of both mutations with our model (Supplementary Fig. 3d and e).

### EZH2 depletion promotes resistance in K562 cells

**Figure 4:**
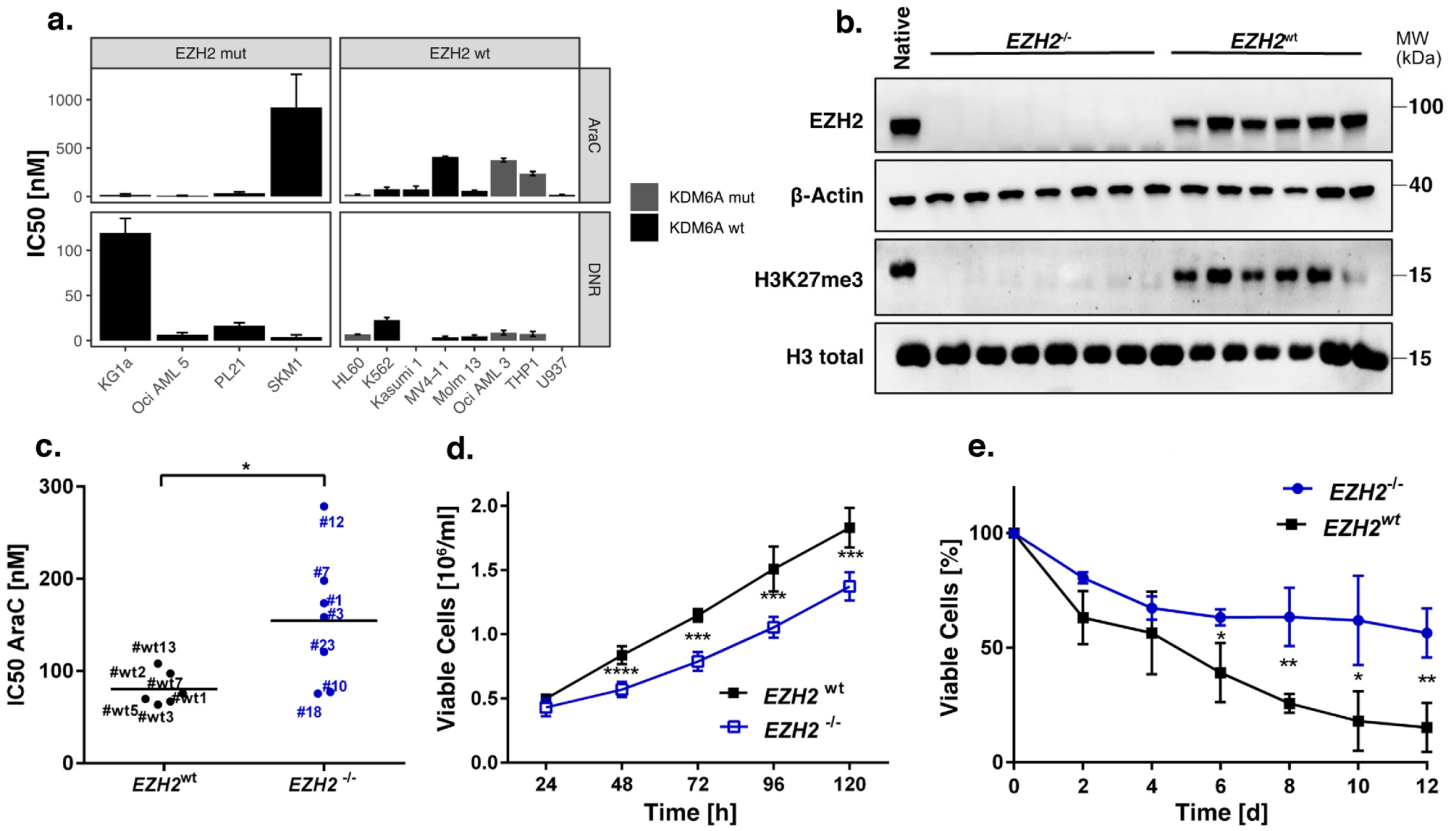
EZH2 depletion promotes resistance in the myeloid cell line K562. **(a)** Comparison of IC_50_ values for DNR and AraC in twelve haematopoietic cell lines. Cells were treated with AraC/DNR for 72 h. **(b)** Immunoblot for EZH2 expression and global H3K27me3 of seven *EZH2*^*−/−*^ and six *EZH2*^*wt*^ sc clones in K562 cells. MW, molecular weight; β-actin and H3 total, loading controls. **(c)** Comparison of AraC IC_50_ values in *EZH2*^*wt*^ (n = 6) and *EZH2*^*−/−*^ (n = 7) clones. Cells were treated with AraC/DMSO for 72 h. Each value represents the mean of three independent experiments. **(d)** Proliferation of *EZH2*^*wt*^ (n = 4) and *EZH2*^*−/−*^ (n = 7) clones for 5 d. Medium was changed every 48 h. **(e),** Long-term low dose AraC treatment in *EZH2*^*wt*^ (n = 3) and *EZH2*^*−/−*^ (n = 3) clones. Cells were treated with 30 nM AraC/DMSO for 12 d. Viable cells relative to untreated control. Unpaired, two-tailed Student’s t-test; *p < 0.05; **p < 0.01; ***p < 0.001. Error bars indicate mean ± s.d of three independent experiments.

In order to study the impact of *EZH2* mutations on chemoresistance in a hematopoietic context, we screened 12 AML cell lines with *EZH2* mutant or wild type background (Supplementary Table 1). We identified two *EZH2* mutated cell lines, SKM-1 and KG-1a, that seem to be more resistant against cytarabine and daunorubicin, respectively (Fig. 4a). Notably, three of the *EZH2*^*wt*^ cell lines were harbouring *KDM6A* mutations, which can also affect drug resistance^42^. Furthermore, we found a strong positive correlation between H3K27me3 levels and EZH2 protein expression (Supplementary Fig. 3a). Next, we established seven *EZH2*^*−/−*^ single cell (sc) knockout clones in the myeloid cell line K562, using CRISPR Cas9 genome editing (Fig. 4b, Supplementary Fig. 4e). Both knockout and control cells were treated for 72 h with either AraC or DNR. In K562/*EZH2*^*−/−*^ clones, increased chemoresistance was found against AraC, while sensitivity against DNR was not affected (Fig. 4c, Supplementary Fig. 4c). Additionally, we observed reduced proliferation in K562/*EZH2*^*−/−*^ clones compared to K562/*EZH2*^+/+^ clones (Fig. 4d). Furthermore, the response towards AraC treatment was studied in a long-term proliferation assay, consequently treating single cell clones for 12 days with a low dose of AraC. In accordance with the short-term assay, also the K562/*EZH2*^*−/−*^ sc clones of the long-term assay displayed higher resistance against AraC (Fig. 4e, Supplementary Fig. 4d).

### EZH2 re-expression sensitises to AraC treatment in K562 cells

**Figure 5:**
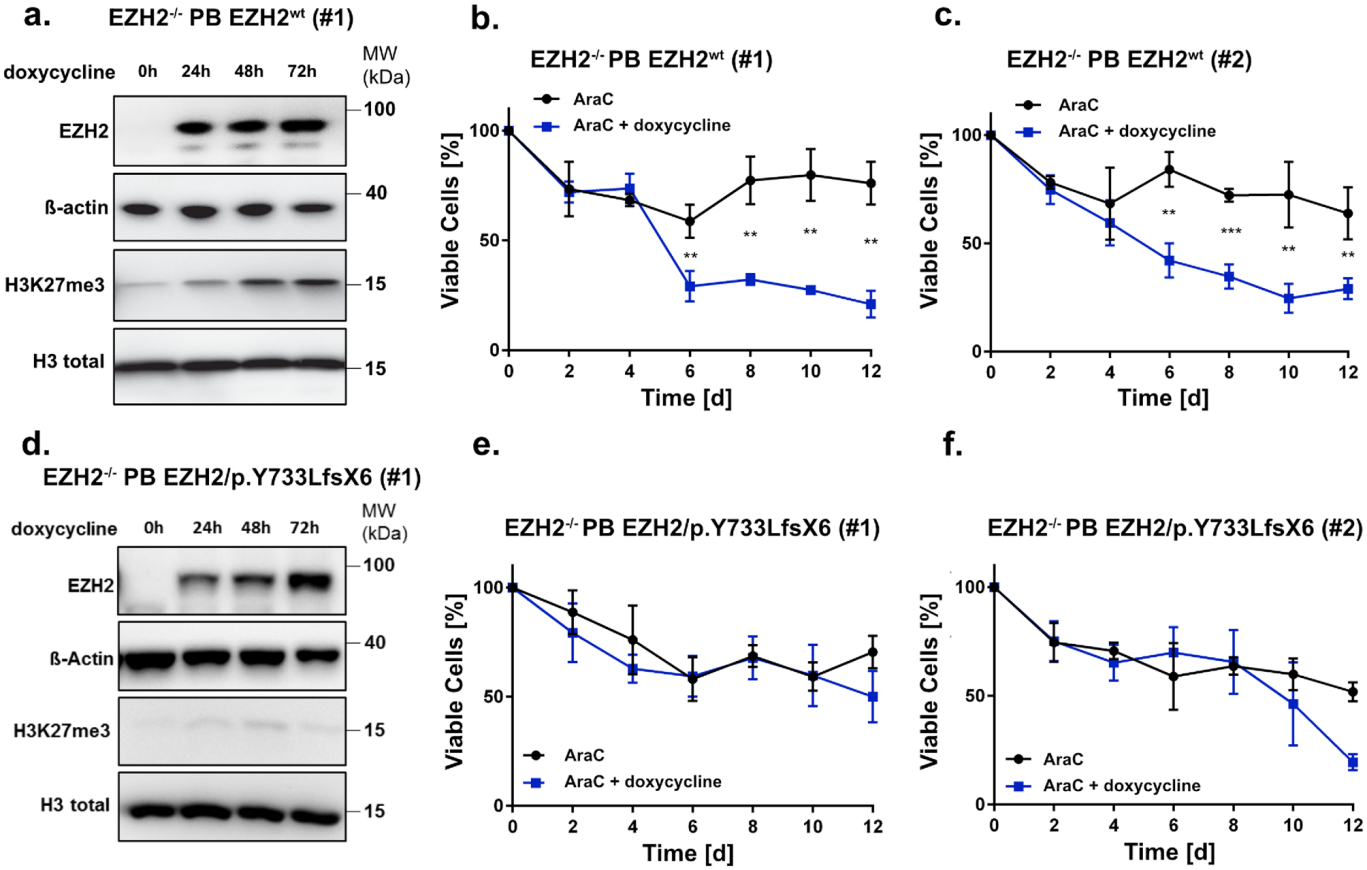
EZH2 re-expression sensitizes K562 cells to AraC treatment. **(a,d)** Immunoblot for EZH2 expression and global H3K27me3 in **(a)** *EZH2*^*−/−*^ PB *EZH2*^*wt*^ cells (clone #1) and **(d)** *EZH2*^*−/−*^ PB EZH2/p.Y733LfsX6 (clone #1) after 0 h, 24 h, 48 h and 72 h of doxycycline induction. Cells were treated with 1 µg/ml doxycycline every 24 h. MW, molecular weight; β-actin and H3 total, loading controls. **(b–c, e–f),** AraC low dose long-term treatment in **(b–c)** *EZH2*^*−/−*^ PB *EZH2*^*wt*^ and **(e–f)** *EZH2*^*−/−*^ PB EZH2/p.Y733LfsX6 cells. Cells were pre-treated for 3 d with doxycycline and then treated with 30 nM AraC/DMSO for 12 d. Cells were split and treated every 4 d and doxycycline was added every 48 h to ensure stable expression of EZH2. Error bars indicate mean ± s.d of three independent experiments. Unpaired, two-tailed Student’s t-test; *p < 0.05; **p < 0.01; ***p < 0.001.

To investigate if re-expression of EZH2 can reconstitute baseline H3K27me3 levels and therefore sensitise cells to AraC treatment, we established a stable, doxycycline-inducible EZH2 expression system via the PiggyBac transposon system (Supplementary Fig. 5a–d). For this reason, DNA coding for *EZH2*^wt^ (AA1-751) was introduced into K562/*EZH2*^*−/−*^ cells (clone #7). Re-expression of wildtype *EZH2* was able to restore global H3K27me3 levels after 48 h (Fig. 5a and Supplementary Fig. 5e). Furthermore, sensitivity against AraC could be restored in a long-term low dose AraC treatment experiment (Fig. 5b,c). Additionally, we introduced DNA coding for the relapse-associated mutation EZH2/Y733LfsX6 (AA1-737, Supplementary Fig. 5c,d). Re-expression of this mutant after doxycycline induction did not result in restoration of H3K27me3 levels (Fig. 5d and Supplementary Fig. 5f). Likewise, the mutation was not able to restore sensitivity against AraC treatment, indicating an involvement of H3K27 trimethylation in the phenotype of chemoresistance (Fig. 5e,f). Doxycycline alone had no effect on the sensitivity of either *EZH2*^*wt*^ or *EZH2*^*−/−*^ cells towards AraC treatment (Supplementary Fig. 5g,h).

### Upregulation of EZH2 target genes desensitises cells to AraC treatment

**Figure 6:**
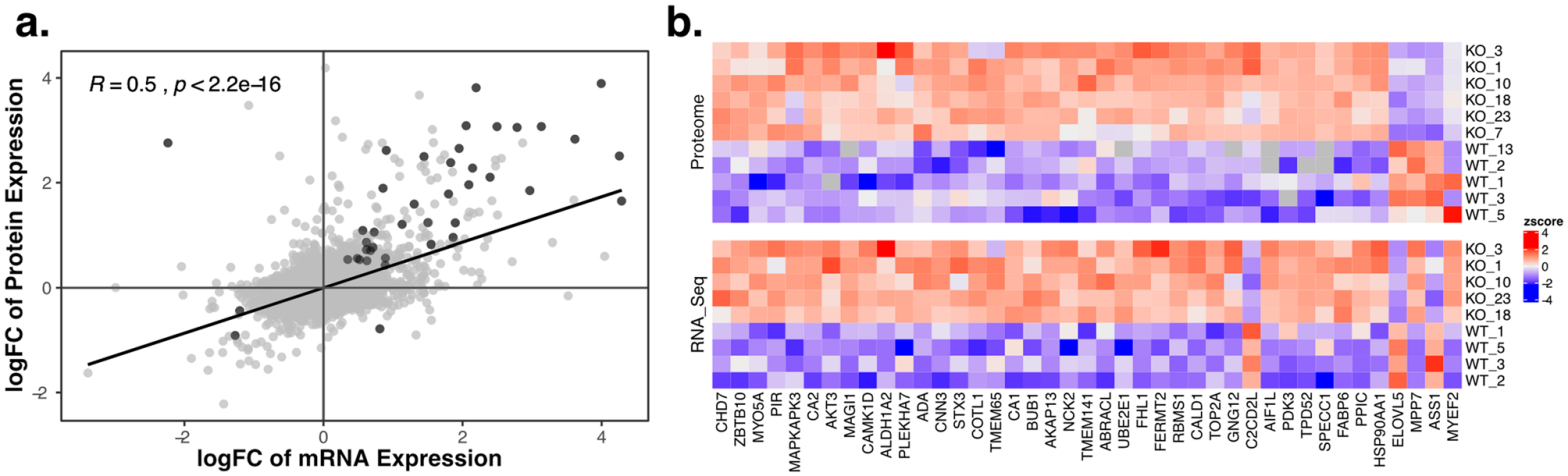
Upregulation of EZH2 target genes. **(a)** Correlation of protein and mRNA expression. Dark grey points representing genes differentially expressed in both measures (adj.P < 0.05). Pearson’s correlation. **(b)** Heatmap of the 41 genes differentially expressed (adj.P < 0.05) between *EZH2*^*−/−*^ and wild type clones, in both protein and mRNA. The colour gradient from red to blue represents high to low expression of genes. White indicates no change.

RNA sequencing and Proteome analysis was performed to uncover the molecular mechanism involved in EZH2-mediated chemoresistance. *EZH2* knockout in K562 cells resulted in aberrant gene and protein expression (Supplementary Fig. 7), visible in transcriptional upregulation of 216 genes and downregulation of 42 genes as well as translational upregulation of 375 genes and downregulation of 205 genes (Supplementary Table 3 and Supplementary Table 4). The change in protein and RNA expression was found to be correlated (R = 0.5, *p* = 2.2e-16, Pearson’s correlation, Fig. 6a) and 41 genes showed differential expression of both mRNA and protein. Most of these genes (37) were upregulated, and only two downregulated in both measures (Fig. 6b). Amongst the upregulated genes, we identified *FHL1* as well as *UBE2E1*, both involved in chemotherapy resistance and relapse in AML^43,44^. Additionally, upregulation of *CA2*, *CNN3* and *AKAP13* was found, which are suggested to be involved in chemotherapy resistance in glioblastoma, colon cancer and breast cancer, respectively. Furthermore, *PDK3*, *TPD52*, *MYO5A*, *AKT3* and *SPECC1* were upregulated, genes associated with poor prognosis in AML or involved in apoptosis. EZH2 ChIP-seq in K562 cells of a publicly available dataset (ENCSR000AQE, ENCSR000AKY) revealed peaks in the promoter region of *FHL1*, suggesting *FHL1* to be a direct target of EZH2. In the promoter region of *UBE2E1* no EZH2 peaks were found, but EZH2 binding was detected in a distal enhancer region (GeneHancer Accession: GH03J023748). Other potential direct targets of EZH2 are *CNN3*, *AKAP13*, *TPD52*, *MYO5A*, *AKT3* and *SPECC1* as EZH2 peaks were found in the respective promoter regions. Additionally, enhancers regulating *FHL1* and *TPD52* could be identified (GeneHancer Accession: GHOXJ136155 and GH08J080078). No peaks were assigned to the genes *CA2* and *PDK3*.

### Resistance of *EZH2* mutated patient-derived xenografts (PDX)

**Figure 7:**
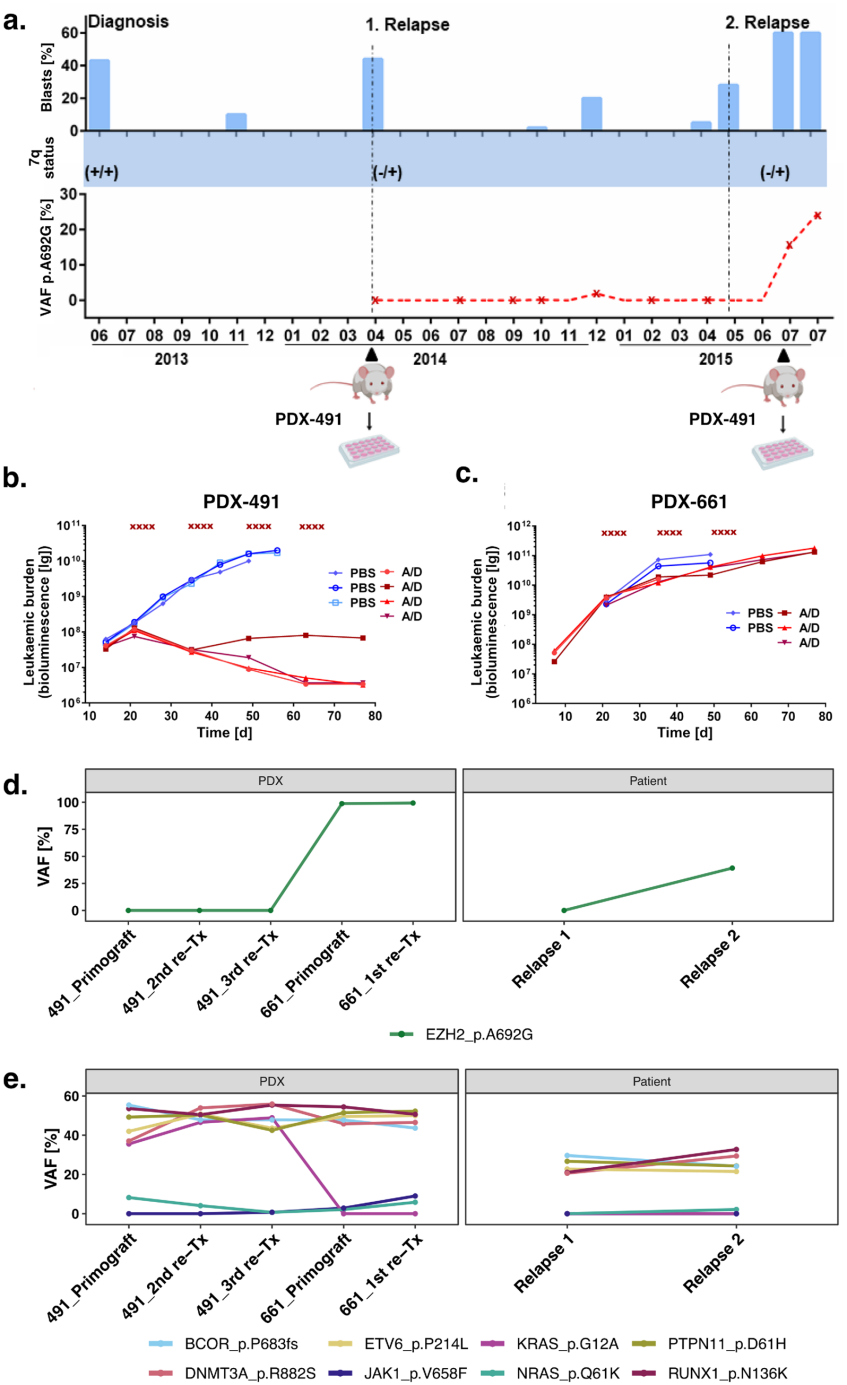
Resistance in an *EZH2* mutated PDX model. **(a)** Course of disease of an AML patient suffering from two relapses (indicated by dashed vertical lines). 7q deletion was confirmed with MLPA. Variant allele frequency (VAF) of the p.A692G mutation was monitored by digital droplet PCR (time points of samples indicated by red stars). Blast count was measured from bone marrow at the indicated time points (blue bars). Samples used for PDX engraftment are indicated with black triangles. In July 2015 two samples were taken in the same month. **(b–c)** In vivo treatment of PDX mice. NSG mice were injected with patient material of relapse 1 and 2, establishing **(b)** PDX-491 and **(c)** PDX-661. 21 d after injection, mice were treated with AraC (100 mg/kg) and DaunoXome (1 mg/kg) (treatment days indicated with red x). Leukaemic burden was monitored in vivo by bioluminescence imaging. Control mice treated only with PBS are shown in blue. **(d–e),** Variant allele frequencies in the course of first to second relapse of **(d)** EZH2/p.A692G and **(e)** other mutations identified by targeted sequencing in Patient and PDX samples. PDX samples of first engraftment (primograft) as well as first (1st re-Tx) second (2nd re-TX) and third (3rd re-TX) re-transplantation.

To extend our findings of *EZH2* associated AraC resistance *in vitro*, we screened relapsed AML samples for clonal outgrowth of *EZH2* mutated cells. We identified a 54-year-old patient who gained an *EZH2* mutation (p.A692G) at second relapse. A summary of the patients’ course of disease including bone marrow blast counts from 9 time points is shown in Figure 7a. We established a sensitive, custom designed digital droplet PCR (ddPCR) assay to monitor the abundance of p.A692G during disease progression from first to second relapse. We detected the mutation only in the second relapse with a more than 20% increase within three months (Fig. 7a). Furthermore, the patient gained a heterozygous 7q deletion at first relapse, as analysed by MLPA (Fig. 7a).

Patient cells of the first and second relapse were serially transplanted into immune-deficient mice, establishing patient-derived xenografts PDX-AML-491 and PDX-AML-661 respectively (Fig. 7a)^45^. Leukaemic cells of the initial diagnosis did not engraft in the model. PDX cells were lentivirally transduced for transgenic expression of luciferase, enabling disease monitoring *in vivo*^36^. We treated the xenograft mice with a combination of cytarabine and daunorubicin and monitored the leukaemic burden over 80 days. We observed a drastic drop in leukaemic cells in the PDX-491 mice, with a complete cure of three out of four animals (Fig. 7b). The treatment of PDX-661 mice only had minimal effect (Fig. 7c).

Targeted sequencing of a panel of 68 recurrently mutated genes^36^ of patient and PDX samples revealed a strong increase of the clone harbouring the *EZH2*/p.A692G mutation in the PDX-661 samples (VAF: 98.8%) in comparison to the second relapse of the patient (VAF: 39.2%, Fig. 7d). The only other mutation illustrating an increase in variant allele frequency was a subclonal *JAK1* mutation, detectable only in the PDX-661 cells. The majority of mutations (*BCOR*, *DNMT3A*, *ETV6*, *PTPN11* and *RUNX1*) remained stable at all time points. Furthermore, two subclonal mutations in *NRAS* and *KRAS* were detected. Both were absent in the patient’s first relapse. KRAS was only detectable in the PDX-491 samples, while NRAS decreased during PDX-491 passaging but was detectable again in the PDX-661 as well as in the patient’s second relapse.

**Figure 8:**
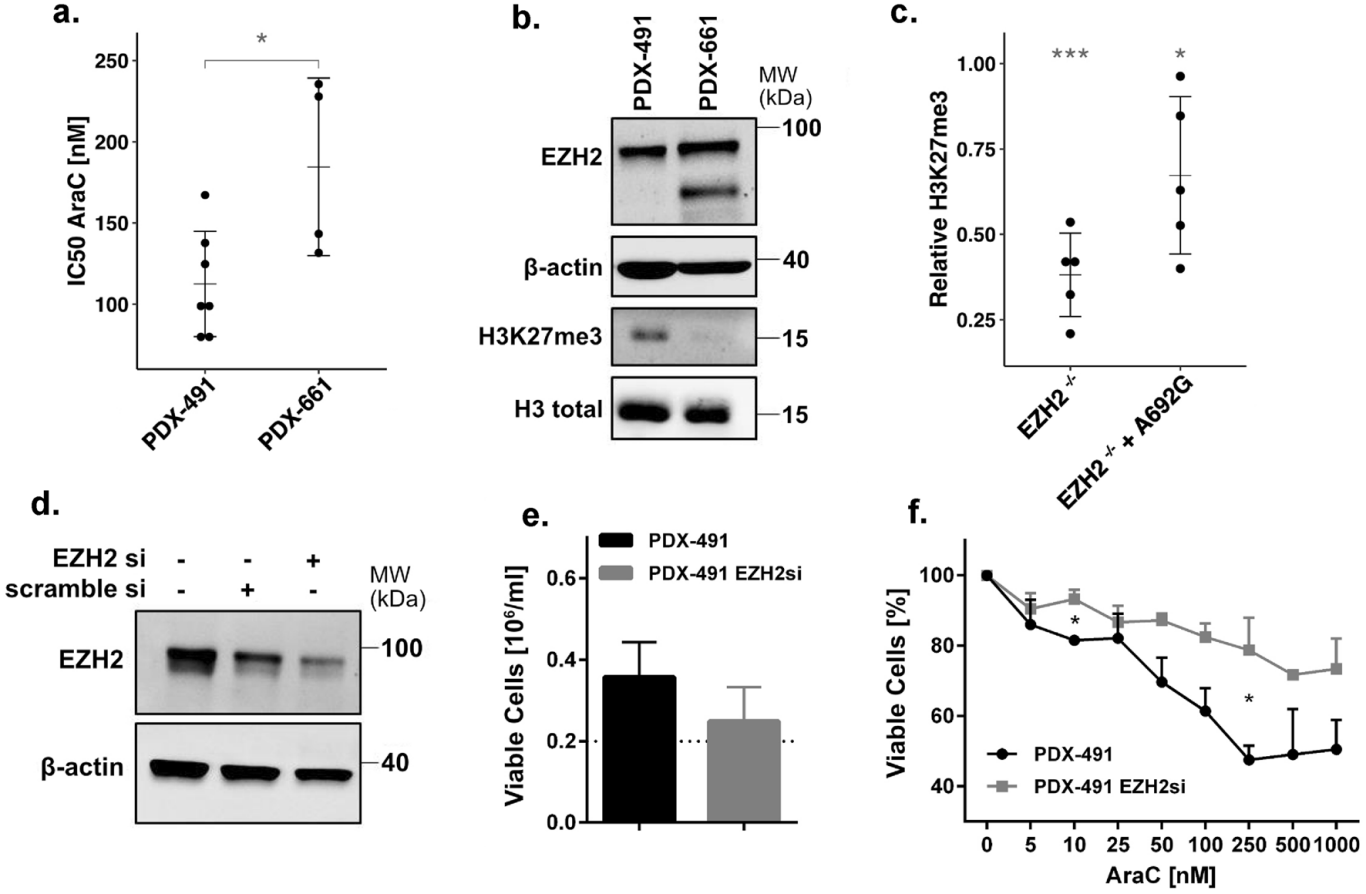
Knockdown of EZH2 in a patient-derived xenograft (PDX) model. **(a)** Comparison of IC_50_ AraC values for PDX-491 and PDX-661 in vitro. **(b)** Immunoblot of EZH2 expression and global H3K27me3 in PDX-491 and PDX-661. **(c)** H3K27me3 levels of EZH2/p.A692G in 293T/*EZH2*^*−/−*^ cells. Values normalized to H3 loading control and relative to wild type. *indicates significant difference to the wild type. **(d)** Immunoblot of EZH2 expression in PDX-491 cells treated with 10 nM siRNA. Representative blot shown for two independent experiments. **(e)** Histogram showing the proliferation of PDX-491 cells with 10 nM siRNA. Cells were pre-treated for 2 d with siRNA and then incubated for another 3 d for the proliferation assay. **(f)** AraC treatment in PDX-491 cells with 10 nM siRNA. Cells were pre-treated for 2 d with siRNA and then treated for 72 h with AraC. Unpaired, two-tailed Student’s t-test; *p < 0.05; **p < 0.01; ***p < 0.001. MW, molecular weight. β-actin and H3 total, loading controls. Error bars indicate mean ± s.d of three independent experiments.

Dose-response analysis of PDX-491 and PDX-661 cells in vitro confirmed an increased resistance of PDX-661 towards AraC (Fig. 8a). Moreover, global H3K37me3 levels were completely depleted in PDX-661 cells, while EZH2 protein expression was stable (Fig. 8b). Transient transfection of the p.A692G mutation into our 293T/*EZH2*^*−/−*^ model revealed decreased global H3K27me3 compared to the wild type, further confirming a LOF phenotype (Fig. 8c). To examine if the observed chemoresistance can be caused by EZH2 depletion, we established an siRNA knockdown (kd) targeting wild type *EZH2* in the PDX-491 cells. EZH2 levels could thereby be reduced by approximately 40% (Fig. 8d). Treatment of these cells for 72 h with AraC resulted in lower proliferation (Fig. 8e) and an increased resistance (Fig. 8f).

## Discussion

Development of resistance against standard chemotherapeutics is common in AML and can be induced through various mechanisms^46^. In this study we report that loss-of-function mutations in the histone methyltransferase EZH2 are associated with increased resistance against the antimetabolite cytarabine (AraC).

*EZH2* mutations in AML are a very rare event. In fact, only 4% of our patients harboured these mutations at diagnosis, with the majority located in the catalytic SET domain, a known hotspot for *EZH2* mutations^28,47^. Seemingly, the mutations induce loss of EZH2 function, independent of the type of mutation. (Fig. 3, Supplementary Fig. 3a). Apart from the SET domain (aa 605-725), also the post-SET domain (aa 725-746), which is essential for the formation of the cofactor S-adenosyl-L-methionine (SAM) binding pocket, was found crucial in maintaining enzymatic function^48^. Two of the mutations identified in our patients, D730_delinsX and Y733LfsX6, previously described in myelodysplastic syndromes (MDS)^49^, caused almost complete elimination of the post-SET domain, while two others, I744fs^50,51^ and G743fs, caused frame shifts that resulted in elongated protein variants, highlighting the importance of this domain. In contrast, the missense mutation K574E, located in the CXC domain, is likely to impair the domain's binding ability to the substrate nucleosome and thereby bringing the H3 tail out of reach^50^.

We found that complete loss of EZH2 promotes AraC resistance in HEK293T cells as well as the myeloid cell line K562 (Fig. 3, Fig. 4). Furthermore, increased resistance was observed in K562 cells expressing the LOF mutation Y733LfsX6 (Fig. 5d–f) and in a PDX model of a patient who gained the LOF mutation A692G at second relapse (Fig. 7). Additionally, low *EZH2* mRNA expression correlated with poor overall and relapse-free survival (Fig. 1b–c). Our findings are therefore in concordance with the study of Göllner et al.^34^, who described AraC resistance in a shRNA knockdown of EZH2 in MV4-11 cells. However, elevated *EZH2* expression has also been reported in AML patients^52,53^, and dual inhibition of EZH1/2 was found to eliminate quiescent leukaemic stem cells (LSCs) to prevent relapse^25^. These combined findings suggest a dual role of *EZH2* as either tumour suppressor or oncogene. In our matched diagnosis/relapse pairs, EZH2 protein and mRNA expression levels were found to be highly patient specific, and in most cases, we observed up- or downregulation in relapse (Fig. 2). EZH2 therefore appears to bear an important function in disease progression, and close monitoring of expression and mutation status seems to be crucial in choosing the best treatment approach.

Interestingly, *RUNX1* and *ASXL1* mutations were significantly co-occurring with mutations in *EZH2*. Similar associations have been described before in myeloid malignancies including AML^28,54,55^. Therapy resistance was associated with frequent co-occurrence of *EZH2* and *RUNX1* LOF mutations^56^, suggesting a cooperative role of these mutations. *ASXL1* LOF mutations on the other hand can establish an additive effect to EZH2 loss by additional reduction of H3K27 trimethylation through inhibition of PRC2 recruitment^29^.

Mutations in other PRC2 subunits (EZH1, EED, SUZ12 or RbAp48) are extremely rare in AML. In the cohort of 50 AML patients of Greif et al. (2018) none could be detected, while in a study of 165 AML patients from Faber et al. (2016), only EED mutations were found with a frequency of 1.8 %. Co-occurrence of *EED* and *EZH2* mutations was found in only one of the patients. EZH2 requires direct interaction with EED to exert its enzymatic function^57^. Thus, also other mutations in the PRC2 complex like EED mutations harbour the potential to confer chemoresistance.

H3K27me3 levels can also be altered by the histone demethylase KDM6A. Loss-of-function mutations in *KDM6A* have been detected in AML and are associated with the development of chemoresistance^42^. Although we and other groups found mutations in both genes to be mutually exclusive^58^, expression levels of KDM6A and EZH2 have an antagonistic effect on global H3K27 trimethylation (Supplementary Fig. 4d). Further research is needed to investigate common and specific EZH2 and KDM6A target sites.

EZH2 is responsible for the trimethylation of H3K27 and therefore inactivation of its target genes. Knockout of *EZH2* in K562 cells induced almost complete loss of H3K27me3 levels and resulted in the upregulation of 216 genes and 375 proteins (Fig. 6b). We identified *FHL1* and *UBE2E1* to be direct targets of EZH2. Overexpression of these genes has recently been described to be involved in resistance against cytarabine, and in relapse in AML patients^43,44^.

FHL1 might be involved in the transmembrane transport of chemotherapeutic agents. Fu et al.^43^ found upregulation of *ABCC1* and *ABCC4*, encoding for the unidirectional efflux transporter proteins MRP1 and MRP4, in AML patients with high FHL1 expression. A slight upregulation of ABCC1 protein could also be detected in our data. Interestingly, Fu et al. also found expression of *FHL1* to be negatively correlated to *SLC29A1* (ENT1) expression. ENT1 is an influx transporter that mediates the uptake of chemotherapeutics and is downregulated upon loss of KDM6A^42^. Since *EZH2* and *KDM6A* mutations were found to be mutually exclusive, those findings suggest an involvement of either EZH2 or KDM6A in the regulation of transmembrane transporter proteins, responsible for the release or uptake of chemotherapeutic agents. The ubiquitin-conjugating enzyme UBE2E1 can regulate the expression of *HOX* genes by its ability to ubiquitinate histones^59^. Although we did not detect any aberrant expression of *HOX* genes, upregulation of HOXA9 as well as HOXB7 was reported by Göllner et al. in a resistant EZH2 negative AML cell line model. We furthermore identified upregulation of the direct EZH2 target genes *CNN3* and *AKAP13*, that are involved in chemotherapy resistance in colon cancer and breast cancer, respectively^60,61^, and the genes *MYO5A*, *AKT3* and *SPECC1*, which are implicated in the evasion of apoptosis^62–64^. Additionally, upregulation of TPD52, involved in proliferation, migration, invasion and apoptosis, was found in many cancer types including AML^65^.

We conclude that loss-of-function mutations in the histone methyltransferase EZH2 have the potential to confer resistance against the chemotherapeutic agent cytarabine and suggest an involvement of upregulated EZH2 target genes in apoptosis, proliferation and transmembrane transport.

## Materials and methods

### Cell culture and patient samples

All cell lines (Supplementary Table 1) were acquired from DSMZ (Braunschweig, Germany) and cultured according to the supplier’s recommendations. Patient-derived xenograft (PDX) AML samples were serially passaged in NSG mice and re-isolated for *in vitro* cultivation as previously described^23,45^. Exclusion of mycoplasma contamination was performed continuously during cell culture using the MycoAlert Mycoplasma detection kit (Lonza, Basel, Switzerland). Analysis of patient samples was based on material of AML patients from the AMLCG-99 trial (NCT00266136), AMLCG-2008 trial (NCT01382147), and the Department of Medicine III, University Hospital, LMU. Mononuclear cells were enriched from bone marrow or peripheral blood by Ficoll density gradient centrifugation. Written informed consent for scientific use of sample material was obtained from all patients. The study was performed in accordance with the ethical standards of the responsible committee on human experimentation (written approval by the Research Ethics Boards of the medical faculty of Ludwig-Maximilians-Universität, Munich, number 068-08 and 222-10) and with the Helsinki Declaration of 1975, as revised in 2000. All animal trials were performed in accordance with the current ethical standards of the official committee on animal experimentation (Regierung von Oberbayern, number 55.2-1-54-2531-95-2010 and ROB-55.2Vet-143 2532.Vet_02-16-7).

### Proliferation assay

Suspension cells were treated with cytarabine (AraC, Selleck Chemicals, Houston, TX, USA), and daunorubicin (in-house). For short time assays, viable cells were treated once (d0) and counted after 72 h on Vi-Cell Cell Viability Analyzer (Beckman Coulter, Krefeld, Germany). For long-term proliferation assays, cells were treated three times (d0, d4, d8) and viable cells were counted every second day. Unpaired, two-tailed Student’s *t*-test and calculation of IC_50_ values were performed using GraphPad Prism version 6.07 (GraphPad Software, La Jolla, CA, USA). PiggyBac^23,45,66^ (PB)/*EZH2* cells were pre-cultured with or without doxycycline (1μg/mL) for 72 h followed by treatment with AraC +/− doxycycline, which was added every 48 h. For knockdown experiments in PDX cells, siRNA targeting EZH2 (#s4918, Thermo Fisher Scientific, Waltham, USA) was transiently transfected (10 nM) via nucleofection (Supplementary Methods). Cells were pre-incubated for 48 h and then treated with AraC for 72 h.

### Immunoblotting

Immunoblotting was performed as described before^3^. The following antibodies were used: anti-EZH2 (#5246, Cell Signaling Technology, Danvers, USA), anti-β-actin (A5441, Sigma Aldrich, St. Louis, USA), anti-H3 (ab1791, Abcam, Cambridge, UK), anti-H3K27me3 (#9733, Cell Signaling Technology, Danvers, USA), anti-SUZ12 (#3737, Cell Signaling Technology, Danvers, USA), anti-RbAP46 (#4522, Cell Signaling Technology, Danvers, USA), anit-EED (ab113911, Abcam, Cambridge, UK), anti-EZH1 (#42088, Cell Signaling Technology, Danvers, USA). Western blots were quantified using ImageJ version 1.50d and levels were normalized to the associated loading control (β-actin for EZH2, total H3 for H3K27me3).

### *In vivo* therapy trial

Patient-derived xenograft (PDX) cells expressing enhanced firefly luciferase and mCherry were established as described previously^45^. For *in vivo* therapy trials, 1*10^5^ PDX-AML-491 or 8*10^5^ PDX-AML-661 luciferase-positive cells were injected intravenously into 11 or 16 week old male NSG mice (NOD.Cg-*Prkdc*^*scid*^* Il2rg*^*tm1Wjl*^/SzJ, The Jackson Laboratory, Bar Harbour, ME, USA), and tumour growth was regularly monitored by bioluminescence imaging (BLI) as described previously^24^. 21 days after transplantation, mice were treated with a combination of Cytarabine (AraC; 100 mg/kg, i.p., days 1-4 of therapy weeks) and liposomal daunorubicin (DaunoXome; 1mg/kg, i.v., days 1 and 4 of therapy weeks) every second week for three (AML-661, n = 3) or four (AML-491, n = 4) cycles. Tumour burden was regularly monitored by BLI and compared to untreated control mice. In total, 13 mice were included in this study; one AML-661 control mouse was sacrificed 14 days after injection due to leukaemia unrelated illness. End point of the study was end-stage leukaemia. All animal trials were performed in accordance with the current ethical standards of the official committee on animal experimentation (Regierung von Oberbayern, number 55.2-1-54-2531-95-2010 and ROB-55.2Vet-143 2532.Vet_02-16-7) and in compliance with the ARRIVE guidelines.

### Ethics approval

We hereby confirm that all experimental protocols were approved by the Department of Medicine III, University Hospital, LMU Munich, the Department of Biology III and Center for Integrated Protein Science Munich (CIPSM); Human Biology and BioImaging, LMU Munich, Planegg Martinsried, Germany and the Helmholtz Zentrum München, Munich*.*

## Supplementary Information

Supplementary Figures.

Supplementary Tables.

## Data Availability

The RNA-seq data generated for this study is available at GEO under the accession number: GSE162623 The mass spectrometry proteomics data have been deposited to the ProteomeXchange Consortium via the PRIDE^67^ partner repository with the dataset identifier PXD023139.
