# Supplementary material for: Loss-of-function mutations in the histone methyltransferase EZH2 promote chemotherapy resistance in AML
Source: Sci Rep. 2021 Mar 12;11:5838. doi: 10.1038/s41598-021-84708-6 (PMC7955088; doi:10.1038/s41598-021-84708-6)

Supplementary Information

LOSS-OF-FUNCTION MUTATIONS IN THE HISTONE METHYLTRANSFERASE EZH2 PROMOTE CHEMOTHERAPY RESISTANCE IN AML

Julia M. Kempf[1,†], Sabrina Weser[1,†], Michael D. Bartoschek[2], Klaus H. Metzeler[1], Binje Vick[3], Tobias Herold[1], Kerstin Völse[3], Raphael Mattes[1], Manuela Scholz[4], Lucas E. Wange[8], Moreno Festini[1], Enes Ugur[2], Maike Roas[1], Oliver Weigert[1], Sebastian Bultmann[2], Heinrich Leonhardt[2], Gunnar Schotta[5], Wolfgang Hiddemann[6,9], Irmela Jeremias[3,6,7] and Karsten Spiekermann[1,6,9,*]

[1]Department of Medicine III, University Hospital, LMU Munich, Munich, Germany. [2]Department of Biology II and Center for Integrated Protein Science Munich (CIPSM), Human Biology and BioImaging, LMU Munich, Planegg Martinsried, Germany. [3]Research unit Apoptosis in Haematopoietic Stem Cells (AHS), Helmholtz Zentrum München, Munich, Germany. [4]Center for Human Genetics and Laboratory Diagnostic (AHC). [5]Biomedical Center and Center for Integrated Protein Science Munich, LMU Munich, Martinsried, Germany. [6]German Cancer Consortium (DKTK). [7]Department of Pediatrics, Dr. von Hauner Children's Hospital, LMU Munich, Munich, Germany. [8]Anthropology & Human Genomics, Department of Biology II, Ludwig-Maximilians-University, Martinsried, Germany. [9]German Cancer Research Center (DKFZ), Heidelberg, Germany.

† These authors contributed equally

* Corresponding author: karsten.spiekermann@med.uni-muenchen.de

Table of Contents

Supplementary Figures	3
Supplementary Methods	10
Supplementary References	15
Uncropped Western Blots	17

Supplementary Figure 1

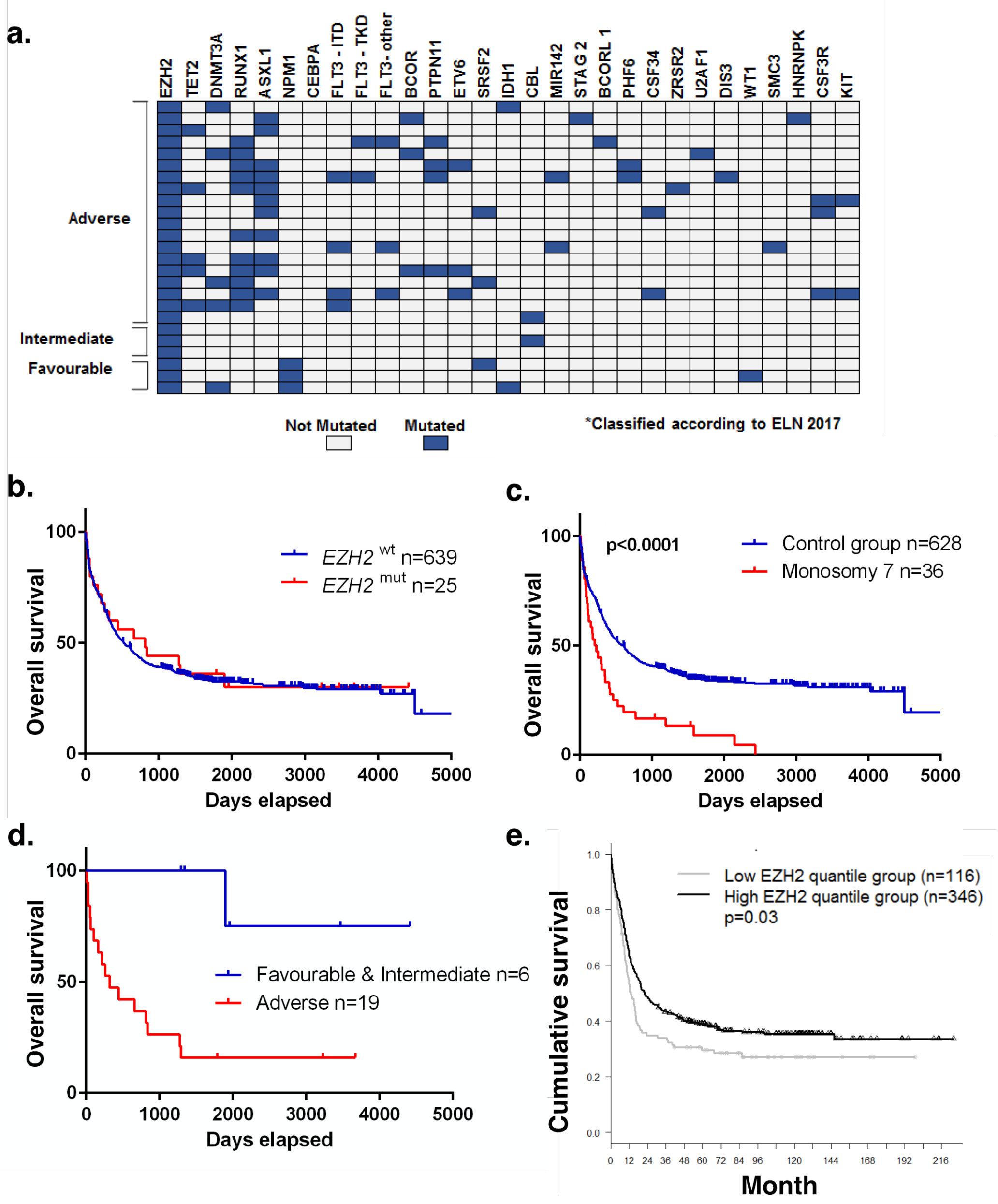

Supplementary Figure 1: Related to Figure 1. Recurrent EZH2 mutations
(a), Mutation pattern of the 25 AML patients harboring EZH2 mutations. Classification according to the ELN- 2017 recommendations. Status also includes multiple mutations in one patient. **(b-d),** Overall survival (OS) of **(b)** patients with EZH2 mutation **(c)** patients with monosomy 7 and **(d)** patients with EZH2 mutation and adverse phenotype (patients from the Metzeler et al. data set). P-value calculated with log-rank test. **(e)** Cumulative overall survival of patients with high or low EZH2 expression of the HOVON dataset (GSE14468). EZH2 high and low groups are defined by the upper and lower quantile. P-value calculated with log-rank test.

Supplementary Figure 2

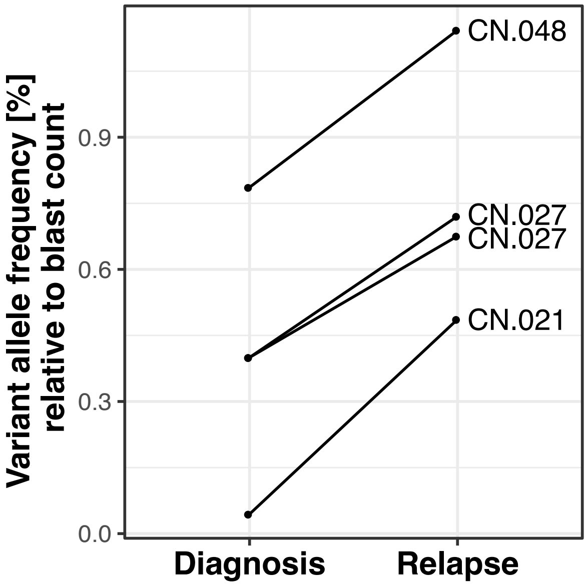

Supplementary Figure 2: Related to Figure 2. Relevance of EZH2 mRNA Expression in AML relapse. Variant allele frequencies (VAF) of EZH2 mutations found in three patients of the Greif et al. cohort. Patient CN.027 harbored two EZH2 mutations. Variant allele frequency was corrected by the respective blast count.

Supplementary Figure 3

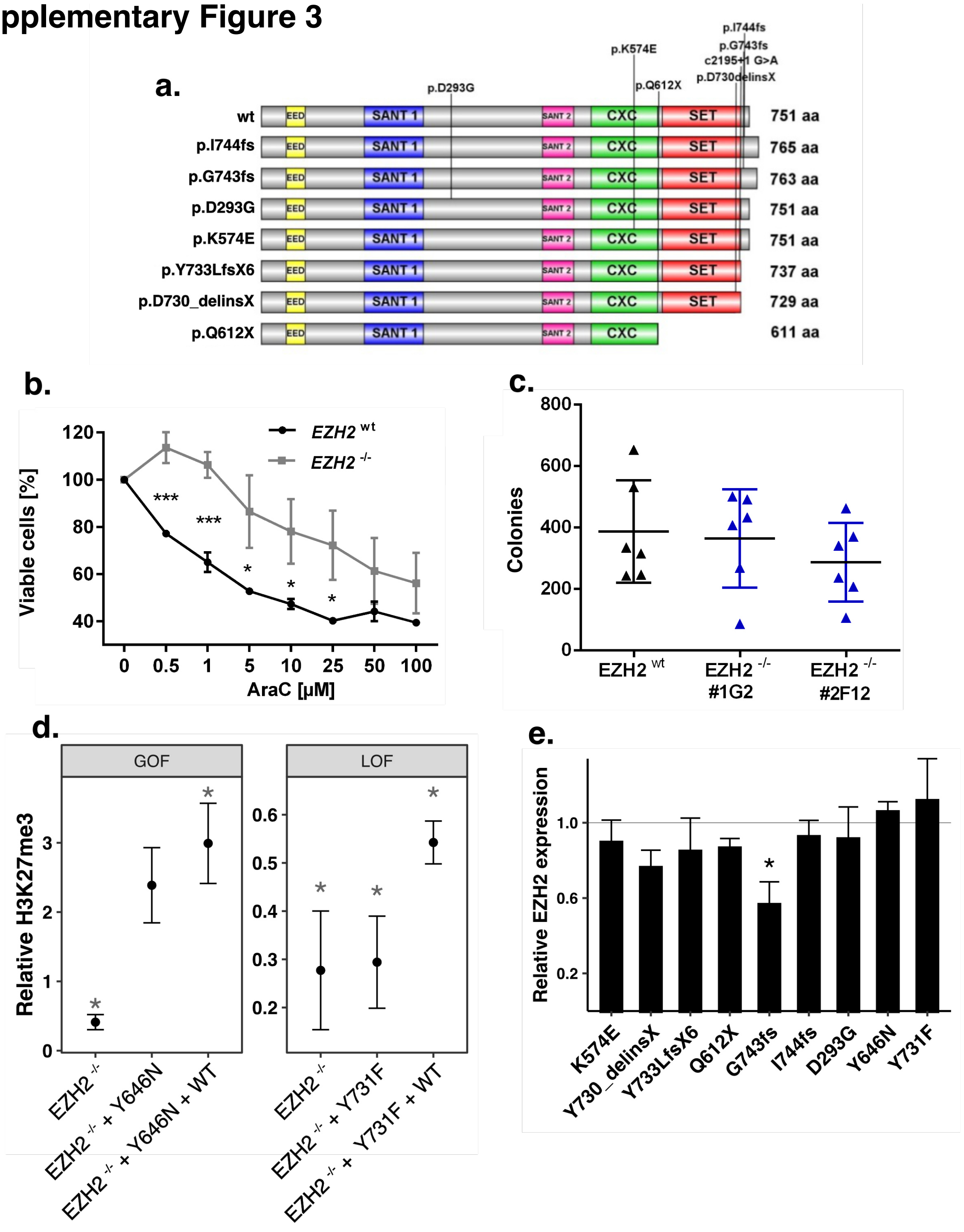

Supplementary Figure. 3: Related to Figure. 3. Evaluation of EZH2 mutations. **(a)** Structural aberrations of investigated EZH2 mutations. **(b)** AraC resistance in one 293T/EZH2$^{-/-}$ and one 293T/EZH2^{wt} sc clone (#1G2, #1D12). Cells were treated for 72h with AraC of different concentrations. Mean for three independent experiments. Viable cells relative to untreated control. **(c)** Colony formation of three 293T sc clones. Mean for 6 independent experiments. **(d)** H3K27me3 levels of the previously described EZH2/p.Y646N and Y731F variants leading to GOF and LOF, respectively. Values relative to WT. Mean for four/three independent experiments. **(e)** EZH2 protein expression of the EZH2 variants. Mean for four independent experiments. Values relative to WT. Unpaired, two-tailed Student's t-test; *p<0.05; **p<0.01; ***p<0.001. Error bars represent standard deviation (s.d).

Supplementary Figure 4

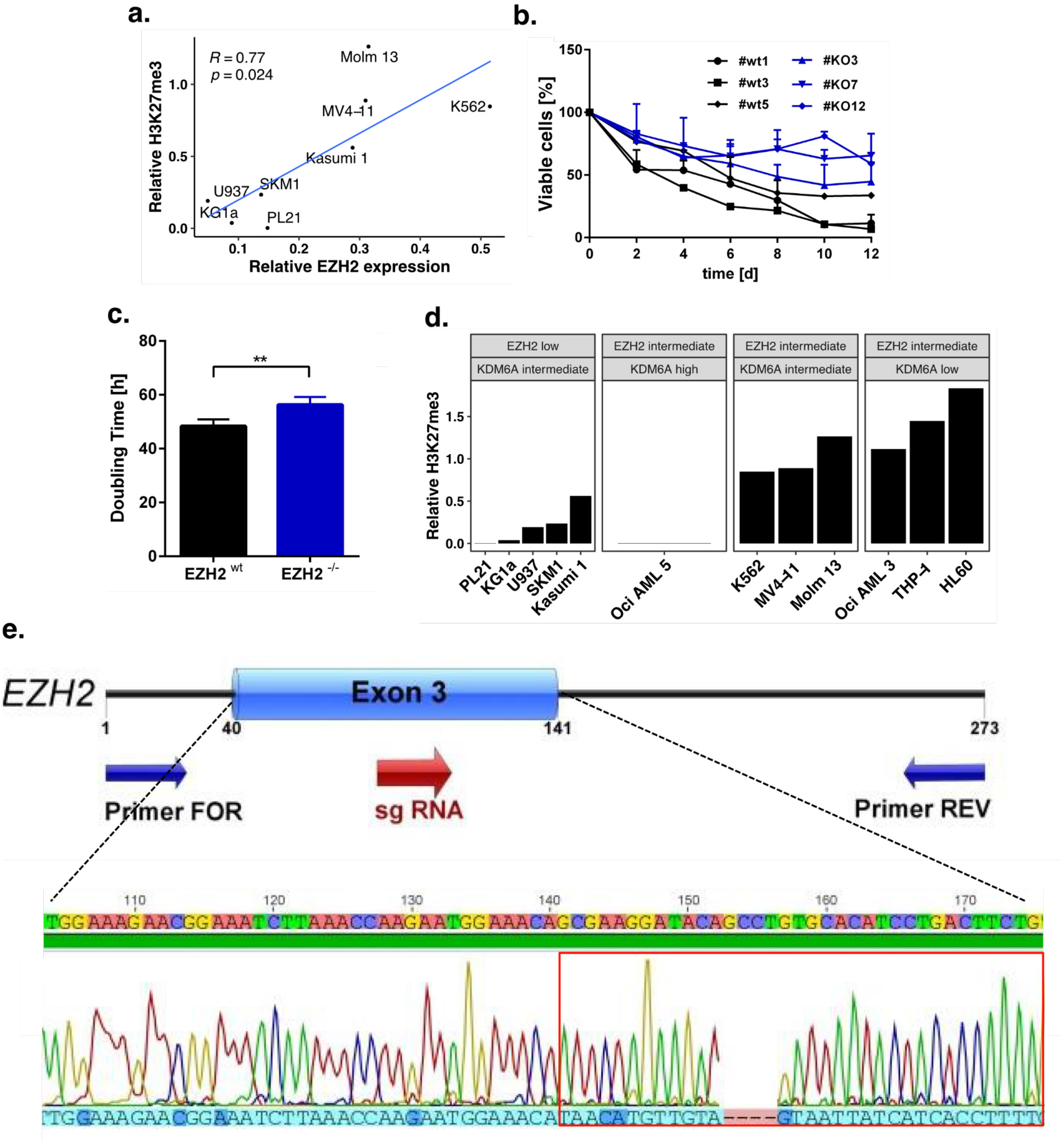

Supplementary Figure 4: Related to Figure 4. EZH2 depletion promotes resistance in the myeloid cell line K562. **(a)** Correlation between EZH2 protein expression and global H3K27me3 in eight hematopoietic cell lines (Pearson's correlation). **(b)** Long-term low dose AraC treatment in $EZH2^{wt}$ (n=3) and $EZH2^{-/-}$ (n=3) clones. Cells were treated with 30 nM AraC/DMSO for 12 days. Cells were split and treated every four days if necessary. Mean ± s.d are given for three independent experiments. **(c)** Doubling time of $EZH2^{wt}$ (n=6) and $EZH2^{-/-}$ (n=7) sc clones. Each clone represents the mean of three independent experiments. **(d)** Combined influence of EZH2 and KDM6A expression on the H3K27me3 levels in the 12 hematopoietic cell lines. Low and High group defined as < 0.3 and > 0.7 relative protein expression. **(e)** Schematic representation of exon 3 Sanger sequencing for CRISPR/Cas9 screening. Mutation is idicated by red box.

Supplementary Figure 5

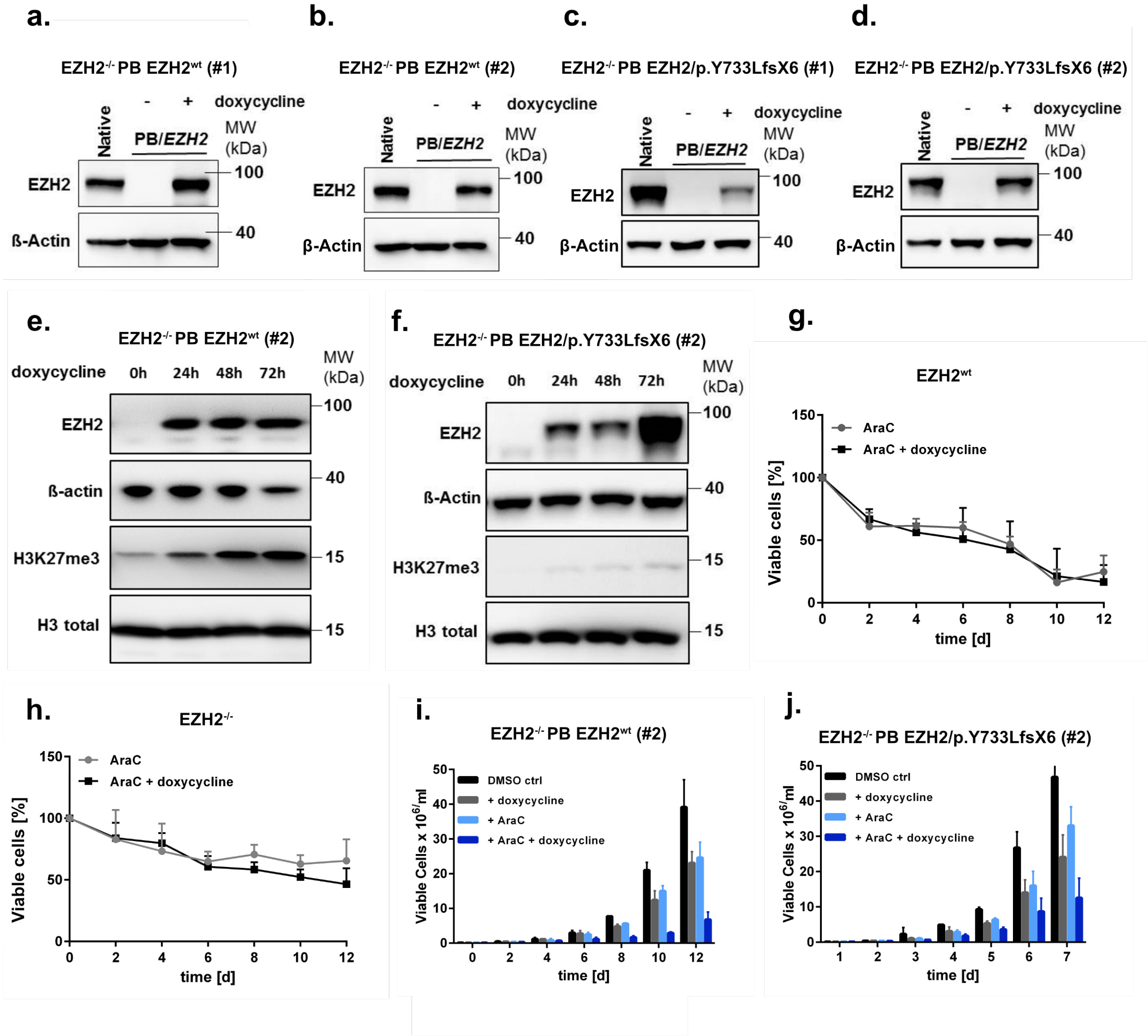

Supplementary Figure 5: Related to Figure 5. Conditional EZH2 re-expression in the myeloid cell line K562 via PiggyBac. **(a-d)** Immunoblot for EZH2 expression with or without doxycycline induction in **(a-b)** EZH2$^{-/-}$ PB EZH2^{wt} and **(c-d)** EZH2$^{-/-}$ PB EZH2/ p.Y733LfsX6 cells. Respective clone given in the plot title. **(e-f)** Immunoblot for EZH2 expression and global H3K27me3 in **(e)** EZH2$^{-/-}$ PB EZH2^{wt} cells (clone #2) and **(f)** EZH2$^{-/-}$ PB EZH2/p.Y733LfsX6 (clone #2) after 0 h, 24 h, 48 h and 72 h of doxycycline induction. Cells were treated with 1 μg/ml doxycycline every 24 h. **(g-h)** Long-term proliferation under AraC treatment (30 nM) with or without doxycycline in **(g)** EZH2^{wt} and **(h)** EZH2$^{-/-}$ cells. Cells were split every 96 h if necessary. Doxycycline was added every 48 h. **(i-j)** Comparison of proliferation with or without indicated treatment and DMSO control in **(i)** EZH2$^{-/-}$ PB EZH2^{wt} and **(j)** EZH2$^{-/-}$ PB EZH2/p.Y733LfsX6 cells. MW, molecular weight; β-actin, loading control. Error bars indicate mean ± s.d. of three independent experiments.

Supplementary Figure 6

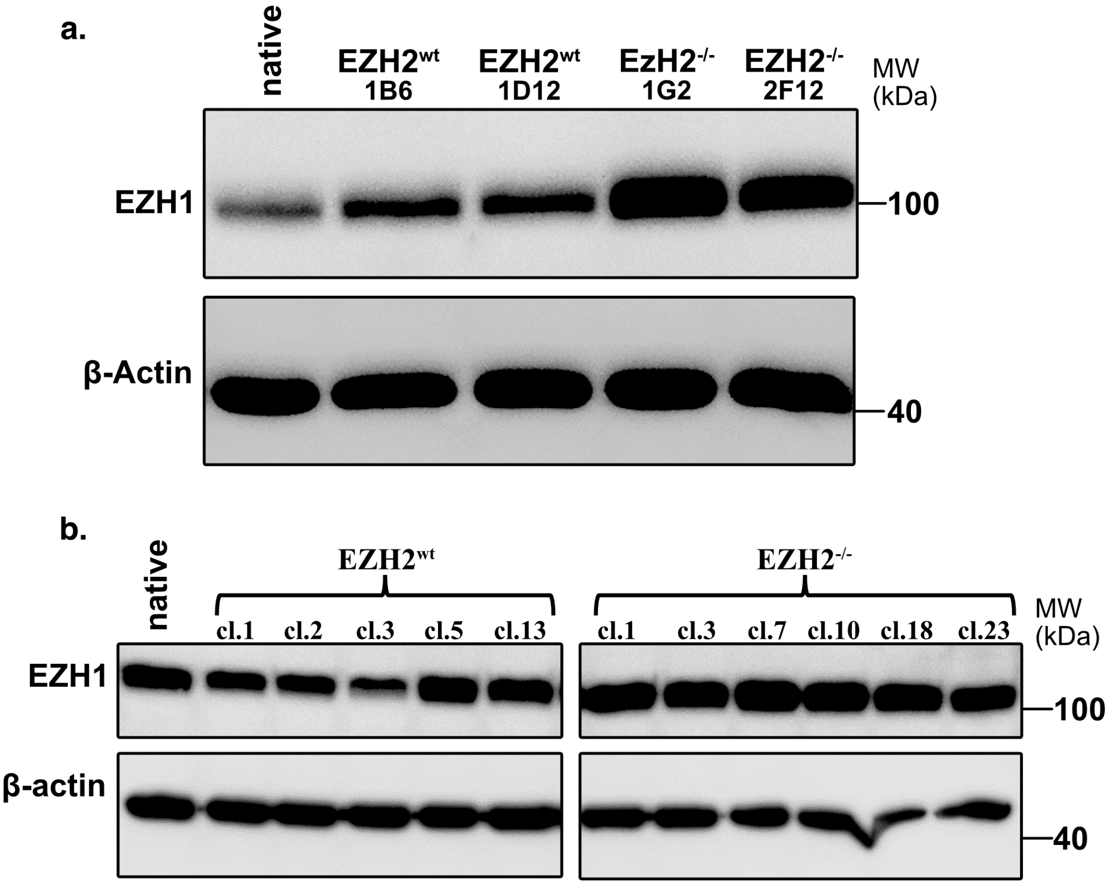

Supplementary Figure 6: Expression of EZH1. (a-b) Immunoblot for EZH1 expression of EZH2^{wt} and EZH2$^{-/-}$ clones in **(a)** HEK293T and **(b)** K562 cells. MW, molecular weight. β-actin, loading control.

Supplementary Figure 7

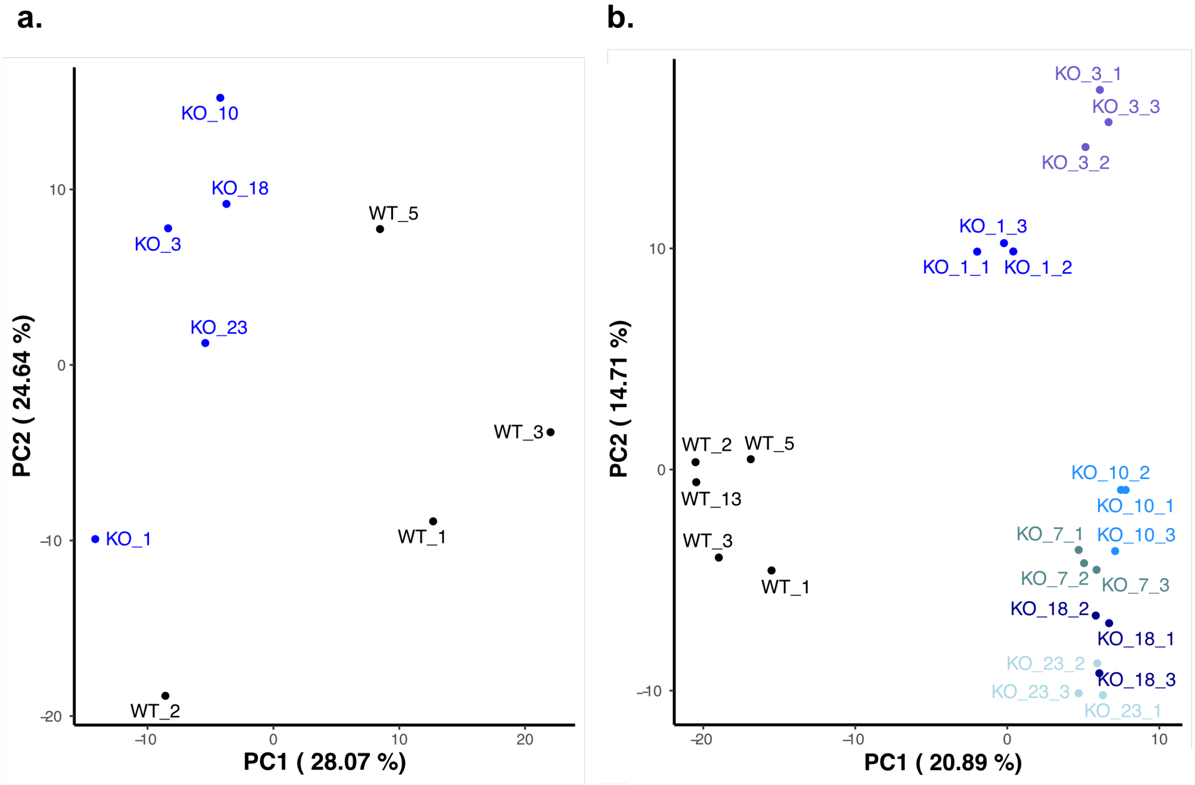

Supplementary Figure 7: Principal component analysis (PCA) of **(a)** RNA-seq and **(b)** full proteome measurements. Altogether five wt K562 clones and six $EZH2^{-/-}$ clones were measured. Additionally, measurement of the proteome was performed in technical triplicates. Technical triplicates cluster closely together, indicating high reproducibility of the measurements. Loss of EZH2 causes aberrant transcript and protein expression, visible in principle component 1 (PC1), the component harbouring the highest variance. PCA on the basis of the 500 most variable genes or proteins. Raw gene count data was normalised with the RLE method in edgeR and scaled to upm (UMIs per million).

Supplementary Methods

Evaluation of global H3K27me3 levels in HEK293T

EZH2 mutant or wildtype constructs were transfected transiently with lipofectamine 3000 (Invitrogen) in 293T/*EZH2*$^{-/-}$ cells. After 72 h, nuclear or whole cell lysates were generated and global H3K27me3 levels were evaluated by immunoblotting.

Transient transfection with siRNA in PDX cells

PDX cells were transfected by electroporation with siRNA using the Neon Transfection System (Thermo Fisher Scientific) with the following parameters: 2200V, 20 ms, 1 pulse. The following siRNA was used: #s4918 EZH2 silencer select (pre-designed by Thermo Fisher Scientific, Waltham, US).

CRISPR/Cas9 mediated genome editing

EZH2 specific sgRNA was cloned into pSpCas9(BB)-2A-GFP (PX458, a gift from Feng Zhang, Addgene #48138). The sgRNA was designed using Benchling (Biology Software, 2018). After 48 h, GFP positive cells were enriched and single-cell sorted into 96-well V-bottom plates (K562) and in 30 cm dishes (HEK293T) with the FACSVantage SE. For HEK293T cells the colonies were separated manually using a 20 µL sterile pipette tip and transferring each cell clone into single wells of a 96-well plate. Cells were cultured at 37°C with 5 % CO_2 for several weeks, until colony formation was observed and sc clones were expanded. To screen for *EZH2* loss, cells were lysed and amplified exon 3 PCR products[1] were sequenced by Sanger sequencing. In short, gDNA was isolated with the QIAamp DNA Blood Mini Kit *(Qiagen, Hilden, Germany)*, in a 96-well plate, re-suspended in 50µL (each well) lysis buffer SC, frozen at -80°C for 30 min, incubated at 56°C for 3h and finally Proteinase K heat inactivated at 85°C for 30 min. 2.5 µL/well of this lysate were directly subjected to PCR mixture (25 µL/rxn., 0.1 µL MyTaq DNA Polymerase) and PCR (FOR: ACAATTTCTCCTTTCCTCTCCTTCA, REV: TGGACACCCTGAGGTCAATGAT) was performed under following conditions: (95°C/5 min- [95°C/30 s - 61°C/30 s - 72°C/30 s] x 45 - 72°C/40 s - 4°C/∞). *EZH2* knockout clones were identified by restriction-fragment length polymorphism (RFLP) analysis of PCR products using HpyAVI. Enzyme recognition site is lost after successful CRISPR/Cas9 targeting. Expression loss was confirmed by immunoblotting for each clone.

PiggyBac/Transposase genome editing

The human *EZH2* fragment was cloned into the BmtI and NotI linearized pPBtet-3xFLAG-IRES-DsRed-Express-PuroR vector[2]. (3xFLAG was removed by linearization) using the In-Fusion HD Cloning Plus Kit (Takara Bio, Saint-Germain-en-Laye, France) generating pPBtet-EZH2-IRES-DsRed-Express-PuroR. For inducible re-expression of EZH2 in *EZH2*$^{-/-}$ K562 cell lines, cells were first nucleofected with equimolar amounts of pPBtet-EZH2-IRES-DsRed-Express-PuroR and PiggyBac transposase-expression vector (Biocat, PB200PA-1). Two days after transfection, cells were subjected to puromycin selection (2 µg/mL) for 3 days. Viable cells were enriched by single-cell sorting into 96-well V-bottom plates with FACSVantage SE (BD Bioscience). Cells were cultured with puromycin (2 µg/mL) until colonies were readily visible. To confirm inducible EZH2 re-expression, clones were treated with doxycycline (1 µg/mL) for a minimum of 24 h and EZH2 expression was analyzed by Western blot.

Generation of inducible PiggyBac *EZH2* cell lines

To generate stable cell lines carrying doxycycline-inducible *EZH2*, cells were first nucleofected with equimolar amounts of pPBtet-EZH2-IRES-DsRed-Express-PuroR and PiggyBac transposase (Biocat, PB200PA-1). Two days after transfection, cells were subjected to puromycin selection (2 µg/mL) for 3 d. Viable cells were enriched by single-cell sorting into 96-well V-bottom plates with FACSVantage SE (BD Bioscience). Cells were cultured with puromycin (2 µg/mL) until colonies were readily visible. To screen for successful EZH2 re-expression, clones were treated with doxycycline (0.5 µg/mL) for 48 h and inducible EZH2 expression was analyzed by Western Blot.

Library preparation and sequencing of K562 clones

RNA sequencing was performed using prime-seq, a bulk version of the single cell RNA-seq method mcSCRB-seq[3]. Briefly, 10,000 cells per sample were lysed in 100 µL RLT Plus Buffer (Qiagen) supplemented with 1 % beta mercapto-ethanol. Following a Proteinase K (Ambion) digest, nucleic acids were isolated using SPRI Beads and a DNAseI (Thermo Scientific) digest was performed on beads. Reverse transcription of the isolated RNA was performed using barcoded oligo-dT primers and template switching oligo. Enabled by the barcoding in the RT, samples were pooled and second strand synthesis as well as amplification of cDNA was performed using Kapa HiFi HotStart polymerase (Roche, Basel,

Switzerland). After quality control of the cDNA using capillary gel electrophoresis (Bioanalyzer, Agilent, Santa Clara, USA), sequencing library preparation was performed using the NEBNext Kit (New England Biolabs, Ipswich, USA). A quarter of the cDNA was used for the fragmentation reaction and a custom adapter was used for ligation. Next, double size selection using SPRISelect beads (Beckmann Coulter, Brea, USA) was performed to only retain fragments between 300 bp and 900 bp. The full step-by-step protocol including primer sequences is accessible at protocols.io (https://www.protocols.io/view/prime-seq-s9veh66). The final library was paired-end sequenced on one HiOut lane of an Illumina HiSeq1500 instrument, with 28 bp in the first read covering the barcode and UMI and 50 bp in the second read covering the cDNA fragment. Raw fastq files were processed using the zUMIs pipeline[4].

Sample preparation for full proteome measurements

Full proteome measurements were performed in technical triplicates for each of the six individual *EZH2*-KOs. In addition, 5 WT K562 cells were measured as a comparison. For each technical replicate 5 x 10^6 cells were lysed in Guanidinium Chloride-based lysis buffer (6 M Guanidinium Chloride, 100 mM Tris-HCl pH 8.5 and freshly supplemented 2 mM DTT). Cell pellets were homogenized by pipetting and boiled for 10 min at 99°C under constant shaking at 1,700 rpm. Quickly after boiling samples were addressed to sonication for 15 min (30 s on/off interval, Bioruptor Plus by Diagenode). To digest the same amounts of protein lysates, the protein concentrations were measured by a BCA assay. Meanwhile chloroacetamide was added to the samples to a final concentration of 40 mM. After at least 20 min of incubation at room temperature 30 µg of each lysate was diluted 1:10 in the digestion buffer (25 mM Tris–HCl pH 8.5 and 10% acetonitrile). Trypsin and LysC were added in a 1:100 protease to protein ratio. Samples were incubated overnight at 37°C and kept under constant shaking at 1000 rpm in a thermal shaker. The next day samples were acidified to stop the digestion with 1% trifluoroacetic acid (TFA), peptide mixtures were subsequently cleaned up on three layers of SDB-RPS[5]. After elution of peptides, samples were dried in a speedvac and resuspended in 20 µL of A* buffer (0.1% TFA and 2% acetonitrile). Prior to LC-MS/MS analysis peptide concentrations were estimated by nanodrop at 280 nm.

Full proteome measurements based on data-independent acquisition method

500 ng peptides of each replicate were separated by nanoflow high-pressure liquid chromatography on an Easy-nLC 1200 (Thermo Fisher Scientific) using in-house packed 50 cm C18 columns (ReproSil-Pur C18-AQ 1.9 µm resin, Dr. Maisch GmbH) and subsequently injected via a nano-electrospray ion source. The peptides were eluted from the column in an acetonitrile gradient for 120 min while the flow rate was fixed to approximately 300 nL/min and the column oven temperature to 60°C. Mass spectrometric analysis of peptides was performed on an Orbitrap Exploris 480 mass spectrometer (Thermo Fisher Scientific, Bremen, Germany).

Data acquisition was performed in data-independent mode (DIA). For this the MS1 resolution was set to 120,000 (mass range of 300 to 1,650 m/z, maximum injection time 60 ms and AGC target 3E6) whereas the MS2 resolution was set to 30k (mass range of 361 to 1,033 m/z, 1 Da window overlap, 30 DIA windows, 1,309 ms cycle time, 22 ms MS2 injection time).

Raw files were processed by the Spectronaut Pulsar X software package (Biognosys, version 14) applying the default Biognosys factory settings for directDIA analysis. As a search basis a reference human proteome (2019) provided by Uniprot was used.

Statistical analysis of Proteome and Transcriptome data

Differential expression analysis was performed in R[6] using limma[7]. For RNA-seq data this was combined with voom[8]. RNA-seq count data was scaled using edgeR[9] and genes with a read count below ten in all samples were excluded. Due to insufficient library size, the samples WT_13 and KO_7 were excluded from the RNA-seq analysis.

MLPA

To identify microdeletions and numerical aberrations in patient samples and PDX cells, the SALSA MLPA MDS Kit from MRC Holland (Amsterdam, Netherlands) was applied. All steps were carried out as recommended by the supplier. 50 ng DNA was used as input and gDNA was generated using the QIAamp DNA Blood Mini Kit (Qiagen, Hilden, Germany). Data were analysed using the ABI 3730 XL sequencer and Genemarker V2.6.0. software from Softgenetics LLC. (State College, US).

Statistical evaluation of Patient Survival

The association of *EZH2* mRNA expression with clinical, genetic and outcome variables was analyzed in publicly available data sets. Patients of the AMLCG1999 trial were used as discovery cohort (GSE37642)[10,11]and results were validated in patients intensively treated in trials of the HOVON (Haemato Oncology Foundation for Adults in the Netherlands) group (GSE14468)[12,13]. Maximally selected rank statistics[14] was used to dichotomize *EZH2* expression in the discovery cohort. The identified cut point was independently validated in the HOVON data set. Kaplan-Meier estimates for overall survival (OS) were calculated using the R survival package with standard parameters. p-values were calculated using the log-rank test. Statistical analysis was performed using the R-3.4.1 software package[6]

Supplementary References

1. Mulholland, C. B. *et al.* A modular open platform for systematic functional studies under physiological conditions. *Nucleic Acids Res.* **43**, e112 (2015).
2. Stief, S. M. *et al.* Loss of KDM6A confers drug resistance in acute myeloid leukemia. *Leukemia* (2019) doi:10.1038/s41375-019-0497-6.
3. Bagnoli, J. W. *et al.* Sensitive and powerful single-cell RNA sequencing using mcSCRB-seq. *Nat. Commun.* **9**, 2937 (2018).
4. Parekh, S., Ziegenhain, C., Vieth, B., Enard, W. & Hellmann, I. zUMIs - A fast and flexible pipeline to process RNA sequencing data with UMIs. *Gigascience* **7**, (2018).
5. Rappsilber, J., Mann, M. & Ishihama, Y. Protocol for micro-purification, enrichment, pre-fractionation and storage of peptides for proteomics using StageTips. *Nat. Protoc.* **2**, 1896–1906 (2007).
6. Team, R. C. & Others. R: A language and environment for statistical computing. (2013).
7. Ritchie, M. E. *et al.* limma powers differential expression analyses for RNA-sequencing and microarray studies. *Nucleic Acids Res.* **43**, e47 (2015).
8. Law, C. W., Chen, Y., Shi, W. & Smyth, G. K. voom: Precision weights unlock linear model analysis tools for RNA-seq read counts. *Genome Biol.* **15**, R29 (2014).
9. Robinson, M. D., McCarthy, D. J. & Smyth, G. K. edgeR: a Bioconductor package for differential expression analysis of digital gene expression data. *Bioinformatics* **26**, 139–140 (2010).
10. Büchner, T. *et al.* Double induction containing either two courses or one course of high-dose cytarabine plus mitoxantrone and postremission therapy by either autologous stem-cell transplantation or by prolonged maintenance for acute myeloid leukemia. *J. Clin. Oncol.* **24**, 2480–2489 (2006).
11. Herold, T. *et al.* Isolated trisomy 13 defines a homogeneous AML subgroup with high frequency of mutations in spliceosome genes and poor prognosis. *Blood* **124**, 1304–1311 (2014).
12. Wouters, B. J. *et al.* Double CEBPA mutations, but not single CEBPA mutations, define a subgroup of acute myeloid leukemia with a distinctive gene expression profile that is uniquely associated with a favorable outcome. *Blood* **113**, 3088–3091 (2009).
13. Taskesen, E. *et al.* Prognostic impact, concurrent genetic mutations, and gene expression features of AML with CEBPA mutations in a cohort of 1182 cytogenetically normal AML patients:

further evidence for CEBPA double mutant AML as a distinctive disease entity. *Blood* **117**, 2469–2475 (2011).

14. Hothorn, T. & Lausen, B. On the exact distribution of maximally selected rank statistics. *Comput. Stat. Data Anal.* **43**, 121–137 (2003).

Uncropped images of western blots

Figure 2a

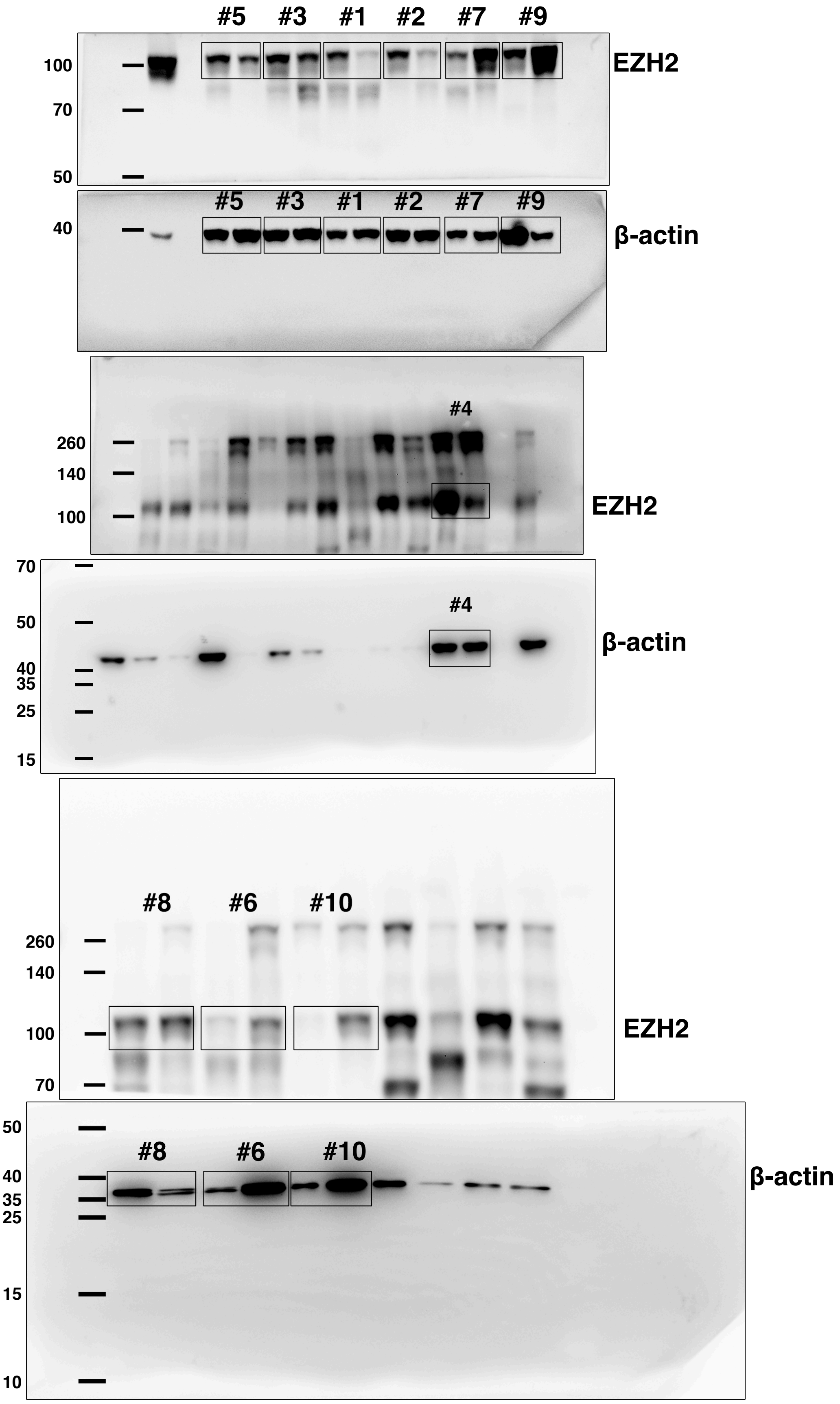

Figure 3a

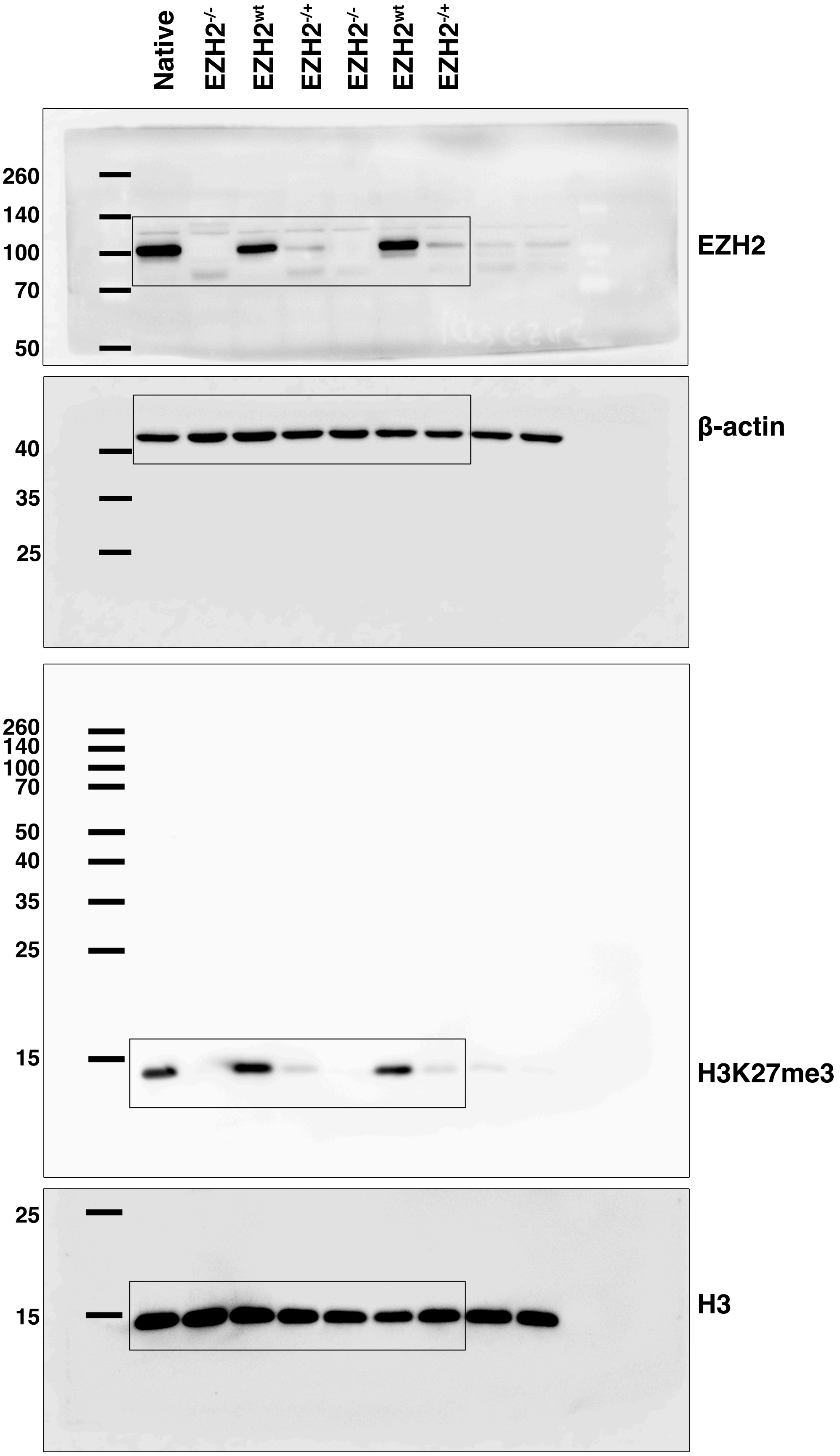

Native
EZH2-/-
EZH2wt
EZH2-/+
EZH2-/-
EZH2wt
EZH2-/+
260
140
100
70
50
EZH2
40
35
25
β-actin
260
140
100
70
50
40
35
25
15
H3K27me3
25
15
H3

Figure 3c

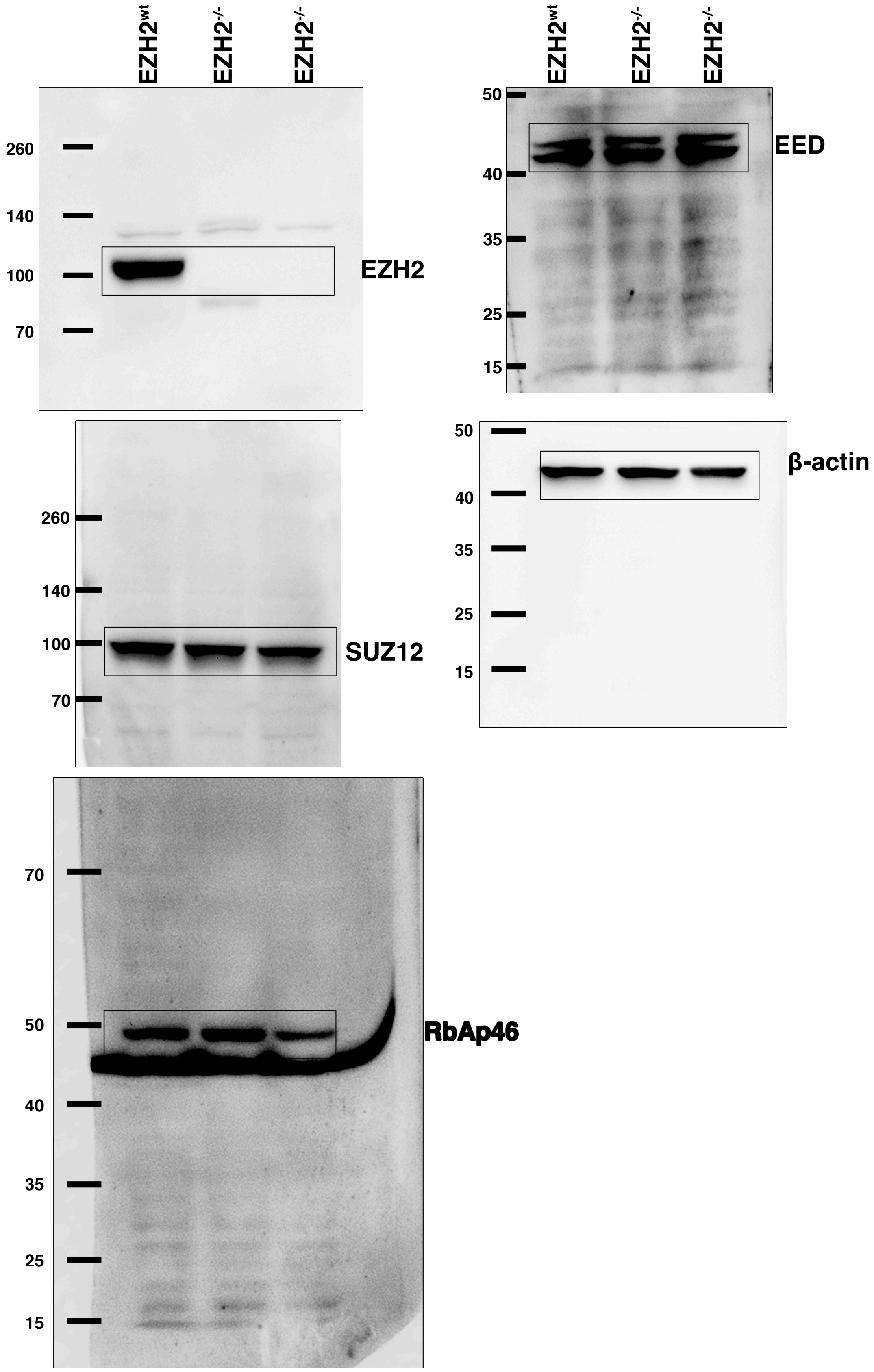

EZH2^{wt}
EZH2$^{-/-}$
EZH2$^{-/-}$
260
140
100
70
EZH2
EZH2^{wt}
EZH2$^{-/-}$
EZH2$^{-/-}$
50
40
35
25
15
EED
260
140
100
70
SUZ12
50
40
35
25
15
β-actin
70
50
40
35
25
15
RbAp46

Figure 4b

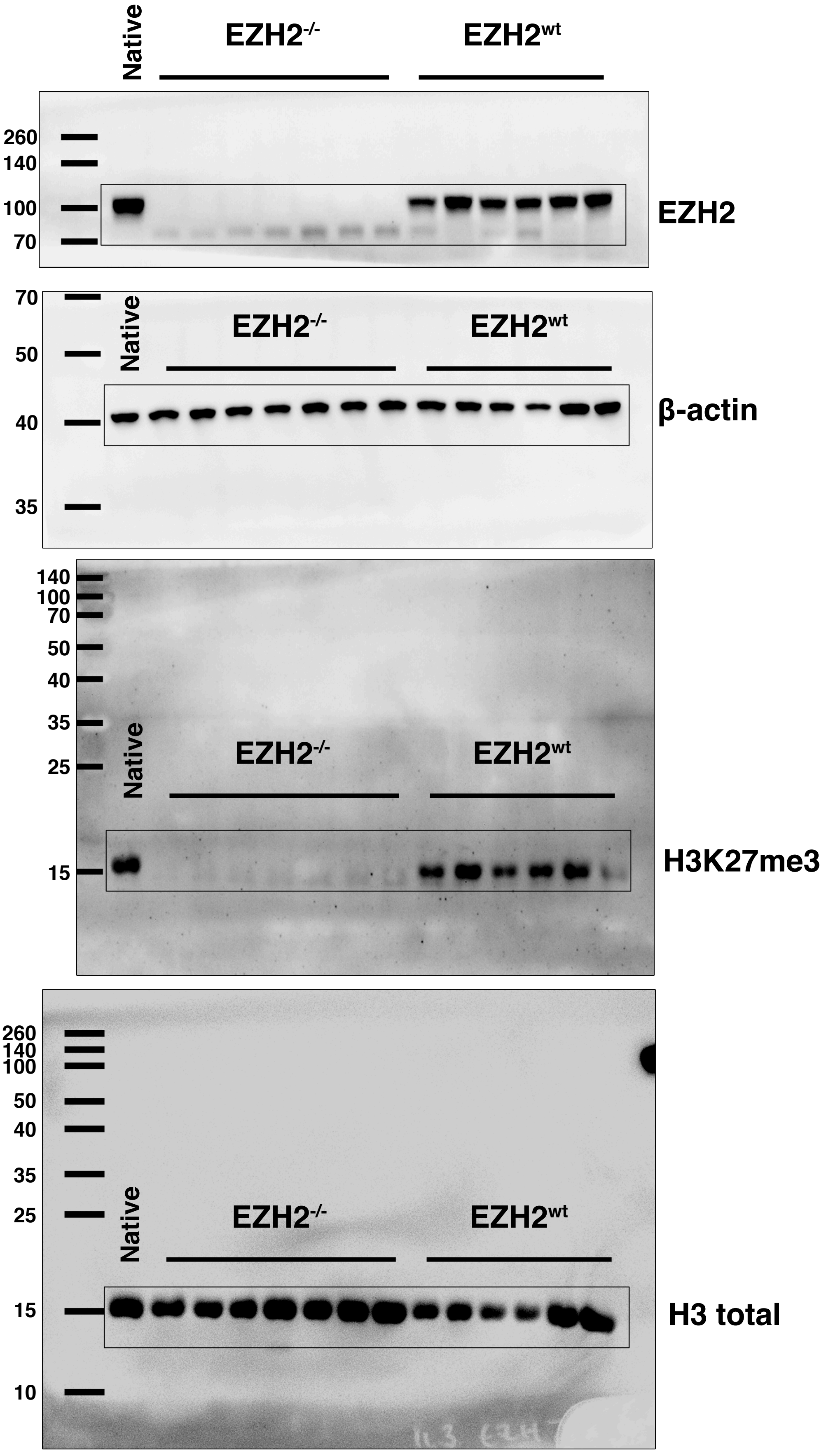
Native
EZH2$^{-/-}$
EZH2^{wt}
260
140
100
70
EZH2
70
50
40
35
Native
EZH2$^{-/-}$
EZH2^{wt}
β-actin
140
100
70
50
40
35
25
15
Native
EZH2$^{-/-}$
EZH2^{wt}
H3K27me3
260
140
100
50
40
35
25
15
10
Native
EZH2$^{-/-}$
EZH2^{wt}
H3 total

Figure 5a & Supplementary Figure 5e

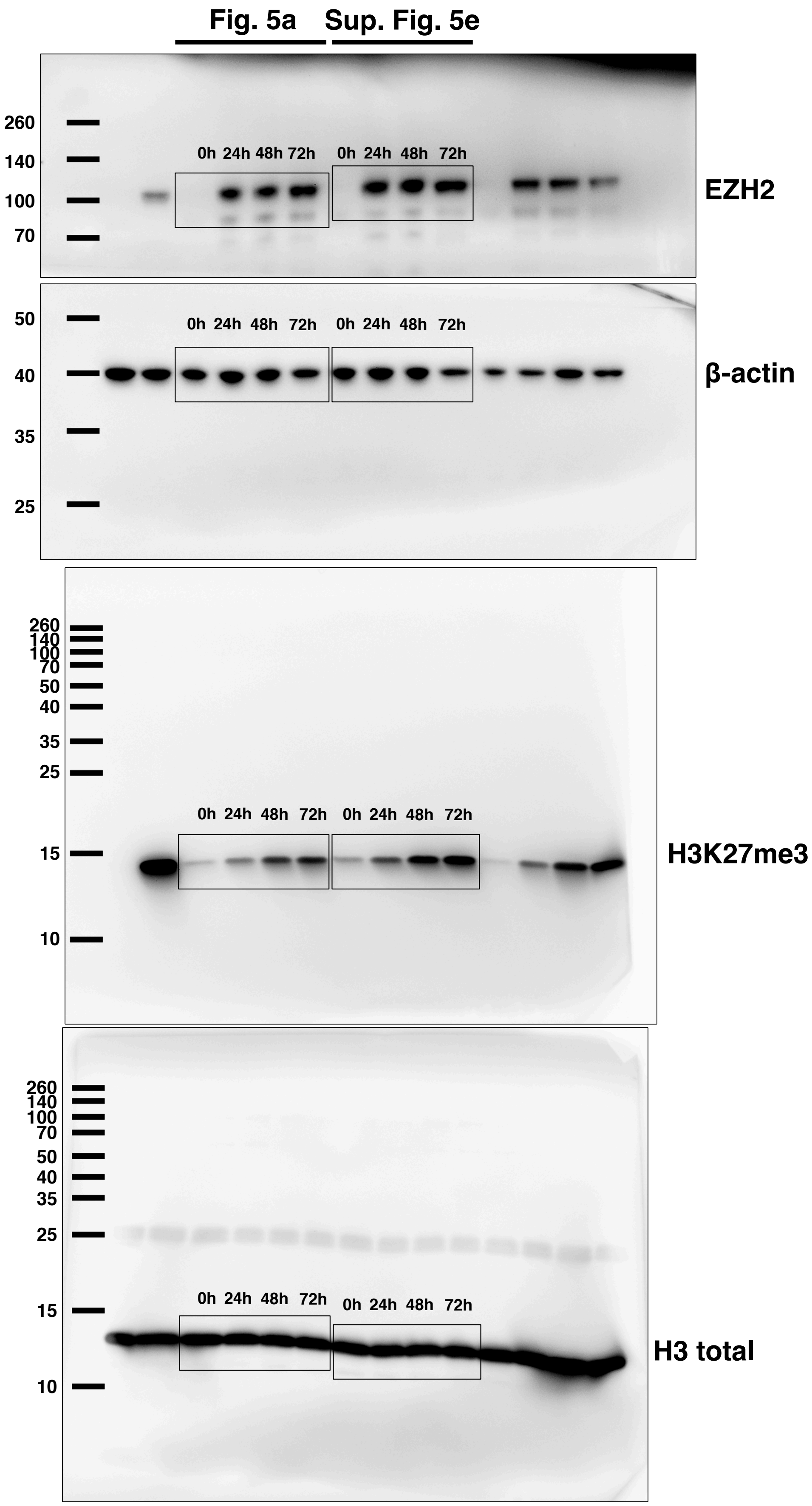

Figure 5d & Supplementary Figure 5f

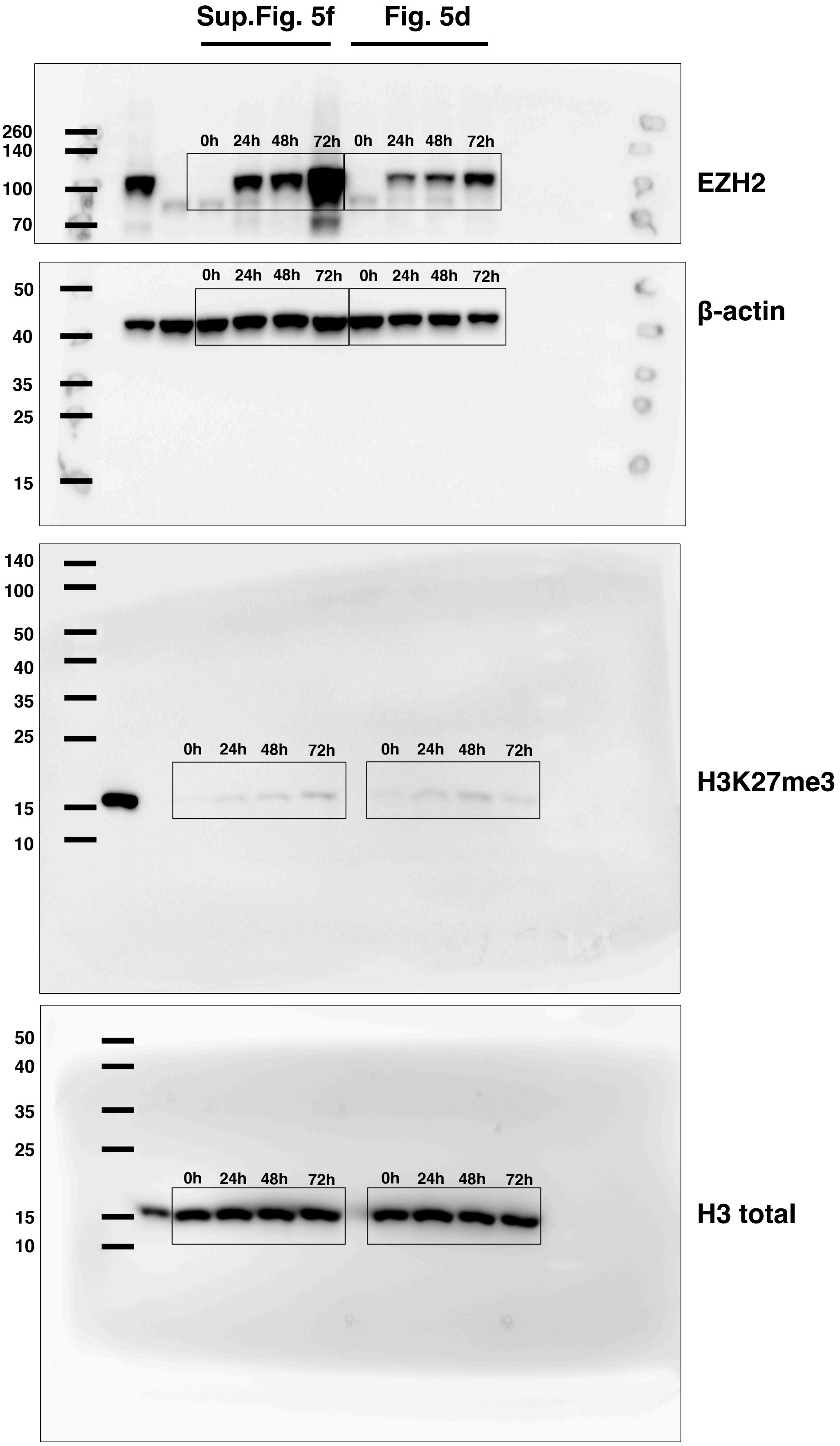

Supplementary Figure 5a & Supplementary Figure 5b

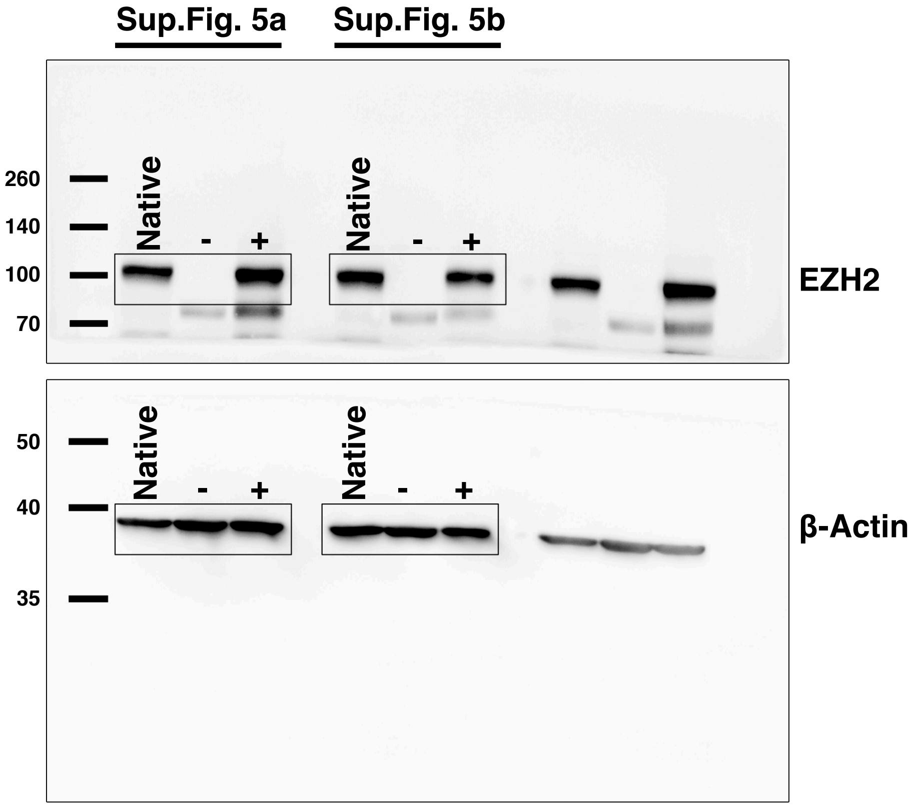

Supplementary Figure 5c & Supplementary Figure 5d

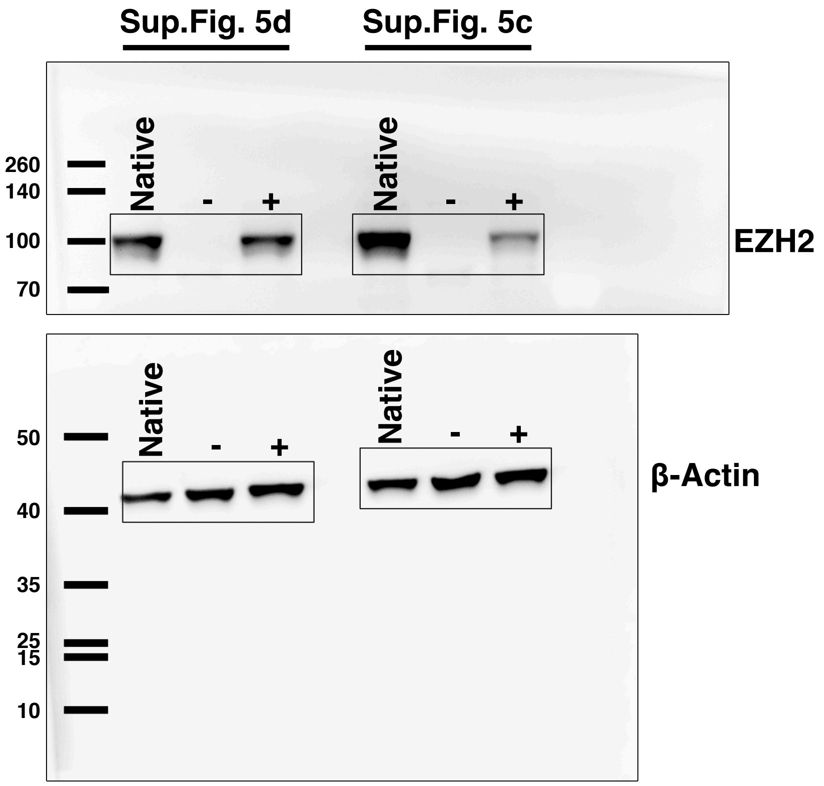

Supplementary Figure 6a

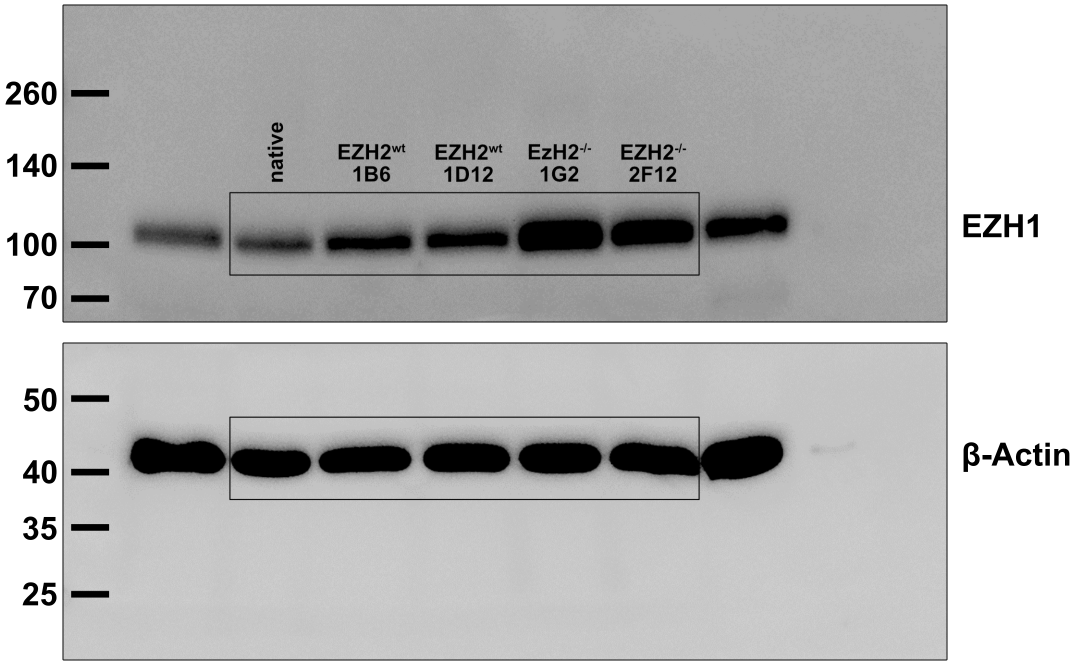

Supplementary Figure 6b

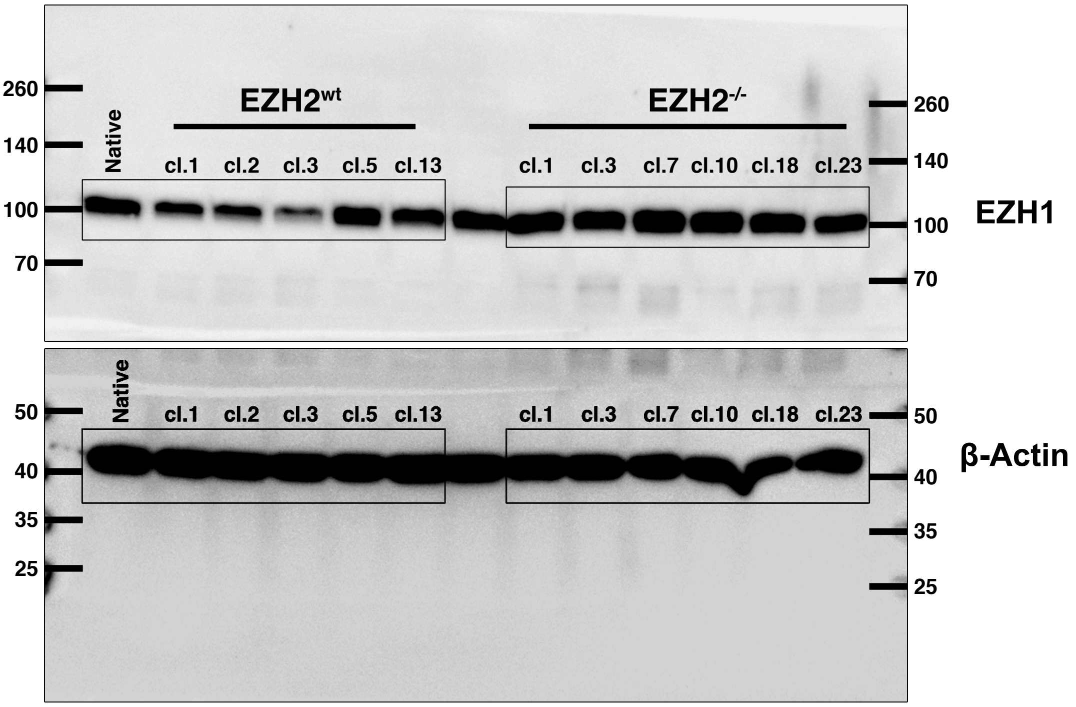

Figure 8b

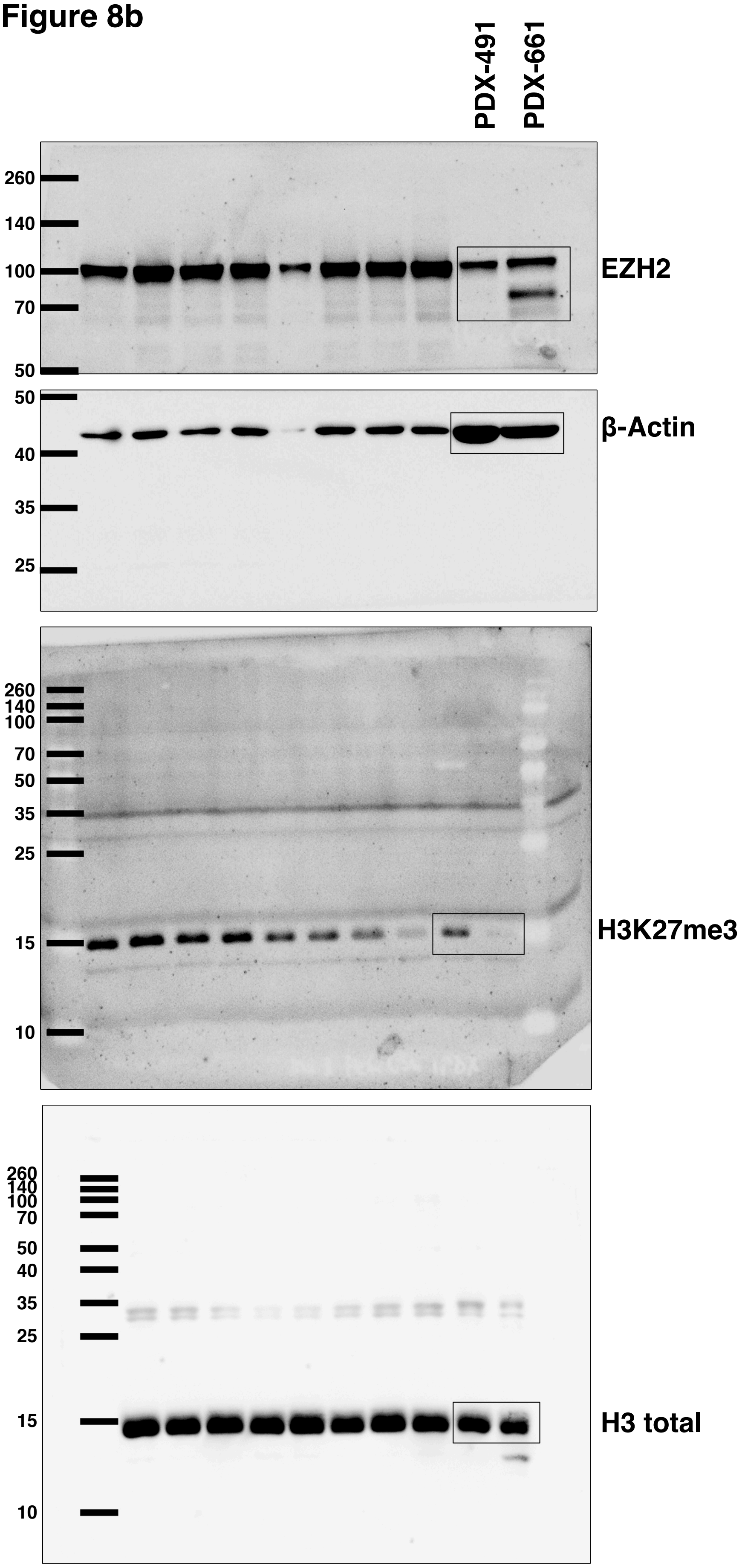
PDX-491
PDX-661
260
140
100
70
50
EZH2
50
40
35
25
β-Actin
260
140
100
70
50
35
25
15
10
H3K27me3
260
140
100
70
50
40
35
25
15
10
H3 total

Figure 8d

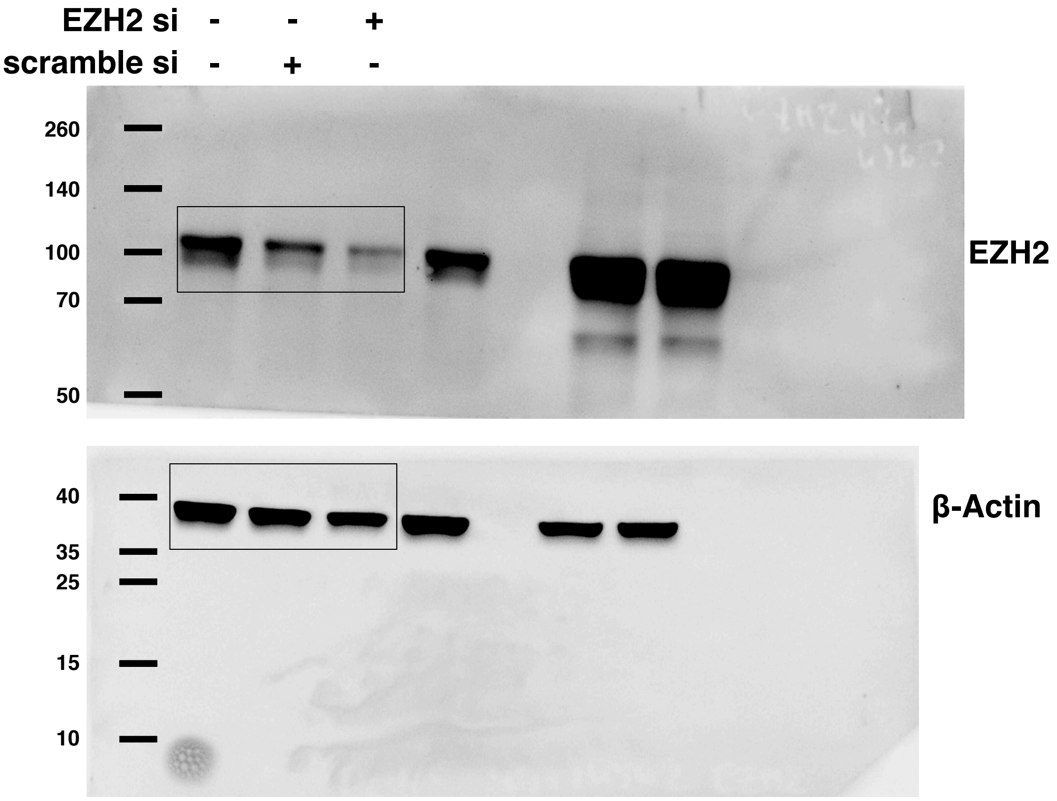

Supplement: Supplementary file 2 — Supplementary Tables. [file 41598_2021_84708_MOESM2_ESM.pdf]
